# Health Impacts of Active Transportation in Europe

**DOI:** 10.1371/journal.pone.0149990

**Published:** 2016-03-01

**Authors:** David Rojas-Rueda, Audrey de Nazelle, Zorana J. Andersen, Charlotte Braun-Fahrländer, Jan Bruha, Hana Bruhova-Foltynova, Hélène Desqueyroux, Corinne Praznoczy, Martina S. Ragettli, Marko Tainio, Mark J. Nieuwenhuijsen

**Affiliations:** 1 ISGlobal, Centre for Research in Environmental Epidemiology (CREAL), Barcelona, Spain; 2 Municipal Institute of Medical Research (IMIM-Hospital del Mar), Barcelona, Spain; 3 Departament de Ciències Experimentals i de la Salut, Barcelona, Universitat Pompeu Fabra (UPF), Barcelona, Spain; 4 CIBER Epidemiología y Salud Pública (CIBERESP), Madrid, Spain; 5 Imperial College London, London, United Kingdom; 6 Center for Epidemiology and Screening, Department of Public Health, University of Copenhagen, Copenhagen, Denmark; 7 Swiss Tropical and Public Health Institute (SwissTPH), Basel, Switzerland; 8 University of Basel, Basel, Switzerland; 9 Kolin Institute of Technology (KIT), Kolin, Czech Republic; 10 Agency for Environment and Energy Management (ADEME), Paris, France; 11 Université de Versailles-Saint-Quentin-en-Yvelines, Versailles, France; 12 The Systems Research Institute (SRI), Warsaw, Poland; 13 UKCRC Centre for Diet and Activity Research (CEDAR), MRC Epidemiology Unit, University of Cambridge, Cambridge, United Kingdom; University of Washington, UNITED STATES

## Abstract

Policies that stimulate active transportation (walking and bicycling) have been related to heath benefits. This study aims to assess the potential health risks and benefits of promoting active transportation for commuting populations (age groups 16–64) in six European cities. We conducted a health impact assessment using two scenarios: increased cycling and increased walking. The primary outcome measure was all-cause mortality related to changes in physical activity level, exposure to fine particulate matter air pollution with a diameter <2.5 μm, as well as traffic fatalities in the cities of Barcelona, Basel, Copenhagen, Paris, Prague, and Warsaw. All scenarios produced health benefits in the six cities. An increase in bicycle trips to 35% of all trips (as in Copenhagen) produced the highest benefits among the different scenarios analysed in Warsaw 113 (76–163) annual deaths avoided, Prague 61 (29–104), Barcelona 37 (24–56), Paris 37 (18–64) and Basel 5 (3–9). An increase in walking trips to 50% of all trips (as in Paris) resulted in 19 (3–42) deaths avoided annually in Warsaw, 11(3–21) in Prague, 6 (4–9) in Basel, 3 (2–6) in Copenhagen and 3 (2–4) in Barcelona. The scenarios would also reduce carbon dioxide emissions in the six cities by 1,139 to 26,423 (metric tonnes per year). Policies to promote active transportation may produce health benefits, but these depend of the existing characteristics of the cities. Increased collaboration between health practitioners, transport specialists and urban planners will help to introduce the health perspective in transport policies and promote active transportation.

## Introduction

Motorized transport is responsible for 70% of environmental pollution and 40% of greenhouse gases emissions in European cities [1]. Multiple national and international organizations such as United Nations Environmental Program, European Environmental Agency and the United States Environmental Protection Agency have proposed transport policies to encourage non-motorized transport (walking and cycling) and public transport in cities to relieve some of the most pressing environmental and health problems [1–3]. While physical inactivity and air pollution have been classified as two of the ten leading risk factors of burden of disease worldwide in 2010 [4;5], transport policies can bring not only benefits from the reduction of car congestion and air pollution emissions, but also important health co-benefits, in particular through an increase in physical activity [6].

Previous studies have quantified the health benefits of replacing car trips with active transportation trips in urban areas [7–10]. These health benefits have been attributed mainly to increased levels of physical activity. However, the majority of previous studies estimated the benefits of physical activity using linear functions which could overestimate the benefits [7–9]. Other factors such as air pollution or traffic accidents have also been considered as risk factors. Only two studies have compared different cities, however these did not consider air pollution as a risk factor for cyclists and pedestrians [10;11].

This is the first study to compare health impacts of active transportation policies across different cities in Europe, providing for the first time an assessment of the effects of various population and environmental risk profiles on health outcomes of active travel. The study includes new and accurate methods to assess the health impacts of physical activity, air pollution and traffic fatalities. The aim of our study was to perform a health impact assessment (HIA) of reaching two active transportation targets (35% cycling and 50% walking) for commuting populations (age groups 16–64) across six European cities (Barcelona, Basel, Copenhagen, Paris, Prague and Warsaw). These targets are based on data from real settings (35% cycling as in Copenhagen and 50% walking as in Paris), where active transportation policies have produced some of the greatest walking and cycling rates in Europe. Furthermore the six cities were chosen to obtain a diversity of active transport policies and characteristics in Europe including cities in diverse geographic regions with different population sizes and densities to allow for comparison. The secondary outcome was a change in emissions of carbon dioxide (CO_2_).

## Methods

### Framework

**Fig 1:**
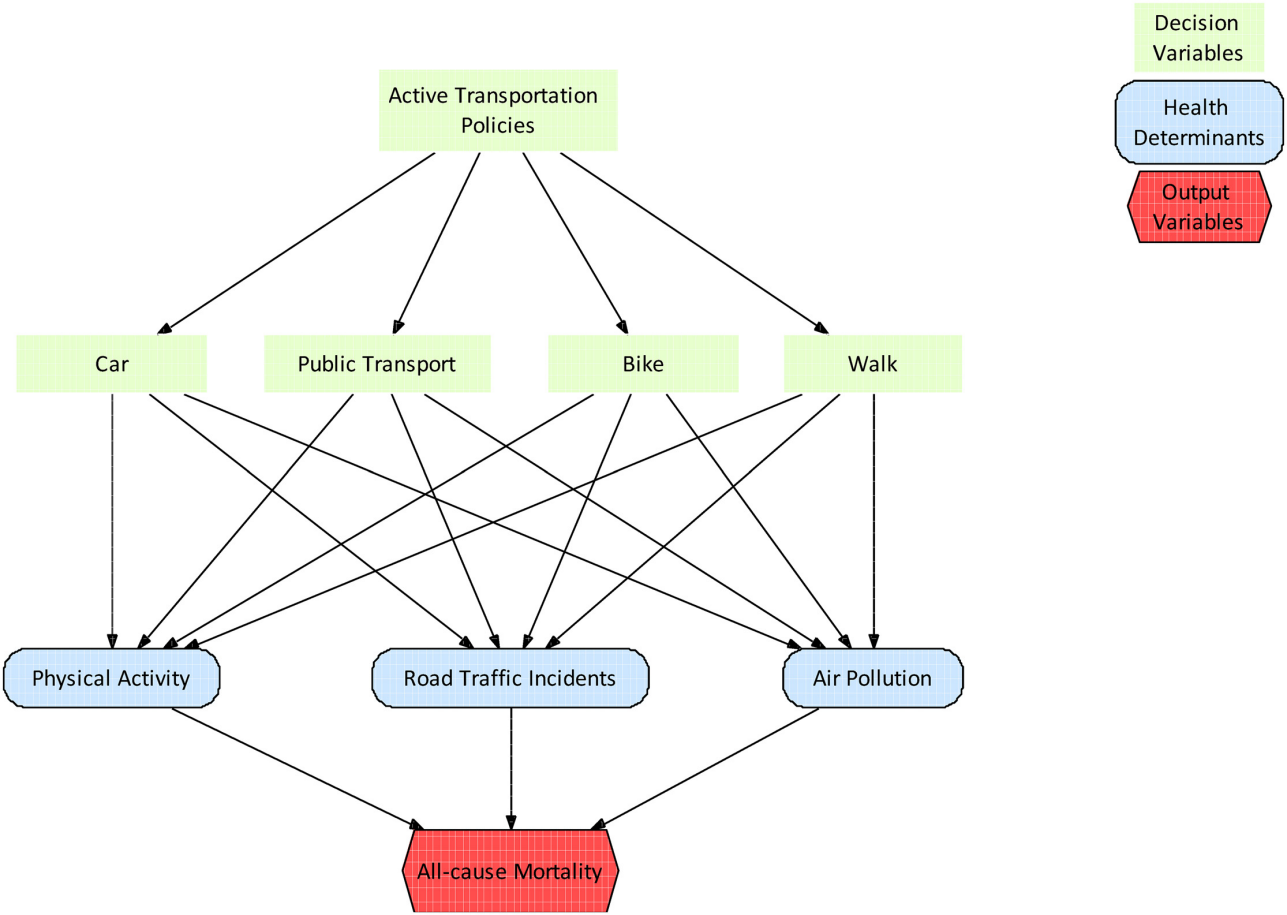
Conceptual framework of active transportation and health.

The conceptual framework for assessing health impacts in travellers is shown in Fig 1. We used a HIA framework as published by Joffe and Mindell [12] to estimate the health effects of mode shifts in our scenarios. We modelled all-cause mortality effects due to physical activity behaviour, road traffic fatalities, and exposure to air pollution [6]. These three health determinants were expected to have the greatest impact and best available data, based on discussions held amongst experts during a workshop in Barcelona in 2009 [6]. We chose all-cause mortality as our main outcome to provide the most robust results possible, given the strongest evidence in the epidemiological literature for mortality. Exposure-response functions were derived from existing meta-analyses to include the best quality of evidence (for air pollution and physical activity) and were calibrated to the current exposure and health conditions in each city. For road traffic accidents we used the traffic safety statistics of each city. Recent publications, such as the health economic assessment tool (HEAT) for walking and for cycling [13], and health impact assessment studies by Woodcock et al. [10], de Hartog et al. [8] and Rojas-Rueda et al. [7;14] provided further guidance to our approach.

We focused the health impacts assessment on city residents who hypothetically would start walking or cycling as a result of the mode shift. In other words, we assessed incremental benefits from physical activity and risks due to increased air pollution inhalation and increase/reduction in exposure to road traffic fatality of new pedestrians and new cyclists, compared to previous exposures. We focused on the age group 16 to 64 years because we expected people from this age group to conduct the majority of trips [15] and to travel regularly to and from work or study, which we assumed were the most likely trips that could be replaced from a daily habit of physical activity behaviour [7].

### Quantitative Model and Data Modelling

The different model parts of the HIA outlined above were linked together in a quantitative model built in Analytica 4.2(Lumina), R 3.0.2(R Project-for-Statistical-Computing), and Excel 2010(Microsoft). The individual models of each exposure (physical activity, air pollution and road traffic fatalities), are described below. A more detailed description of each model is published elsewhere [7;8;10;14] Main input data used in the model are summarized in Table 1. Transport data were obtained and analysed from a combination of data provided by travel surveys and records reported by transport departments of each city, local governments, municipalities, and intergovernmental institutions between 1994 and 2012.

**Table 1:** Baseline data and key assumptions used in the model.

Variable		Barcelona	Basel	Copenhagen	Paris	Prague	Warsaw
**Population in the city** ^a^		1 620 943	164 516	559 440	2 249 977	1 246 786	1 715 517
**Population density (person/km**^**2**^**)**		16 540	6 854	5 800	21 423	2 513	3 318
**City size (Km**^**2**^**)**		98	24	88	105	496	517
**All trips per day (%)**	PT	1 484 788 (30)	443 900 (25)	303 333 (17)	2 027 880 (33)	1 860 517 (50)	2 520 225 (49)*
	Walk	2 302 569 (46)	608 808 (35)	520 615 (29)	2 819 239 (46)	888 383 (24)	997 820 (19)*
	Bicycle	109 282 (2)	265 186 (15)	492 805 (27)	162 147 (3)	9 737 (0.3)	54 818 (1)*
	Car	457 095 (9)	429 320 (24)	491 576 (27)	731 482 (12)	932 643 (24)	1 278 847 (24)*
**Trips per person per day**	All modes	3.1	3.4	3.2	3.4	2.9	3**
**Average distance travelled per trip (km)**	PT	10.0*	13.1	2.8*	7.6	15.7	28.6*
	Walk	1.4*	1.3	0.7*	1.1	1.2	1.1*
	Bicycle	3.3*	2.9	3.7*	3.4	4.4	5.4*
	Car	8.9*	9.5	5.1*	11.4	10.1	20.3*
**Average trip duration (minutes)**	PT	33.2**	44.4	9.3	35.0	33.4	44.0
	Walk	16.2	24.0	9.9	14.0	16.1	17.0
	Bicycle	14.0	14.9	14.0	20.0	29.0	24.0
	Car	24.4	22.8	11.3	28.0	27.9	32.0
**Average speed (km/h)**	PT	18.1	17.7^++^	18.1**	8.4	28.2	39.0^++^
	Walk	5.0	3.3^++^	4.2**	4.4	4.5	3.8^++^
	Bicycle	14.0	11.6^++^	16.0	13.4**	12.0	13.4^++^
	Car	21.8	25.0^++^	27.0	21.7	45.0	38.0^++^
**Road traffic fatalities per year, 16–64 years (Deaths/year)**+	PT	0.0	0.0	0.0	0.0	0.2	2.8
	Walk	11.2	2.0	3.8	16.6	27.6	48.5
	Bicycle	0.2	1.2	2.3	2.7	0.6	2.5
	Car	3.1	0.9	4.6	3.4	5.8	18.8
**Concentration of PM2.5 (μg/m3)**	City annual average	15.6	13.6 ^b^	11.0	18.0	21.0	23.6
	in the Car	35.5	30.9**	25.0**	41.0**	47.8**	53.7**
	in the Bicycle	35.0	30.5**	24.7**	40.4**	47.1**	52.9**
	in the PT	25.9	22.6**	18.3**	29.9**	34.9**	39.2**
	Walking	21.6	18.8**	15.2**	24.9**	29.1**	32.7**
**Expected mortality (deaths/1000 inhabitants)** ^c^	16–64 years	2.05	2.64	2.22	2.73	2.90	3.70
**Deaths per billion of kilometre travelled**	PT ^d^	0.00*	0.00*	0.00*	0.00*	0.02*	0.11*
	Walk	12.87*	9.14*	28.53*	19.08*	70.45*	122.48*
	Bicycle	2.30*	5.63*	3.42*	13.61*	33.05*	23.31*
	Car	2.79*	0.79*	5.04*	1.66*	1.07*	1.99*

Data presented come from local, national or multinational reports and records, including transport surveys, health and air quality reports. The Bicycle data include trips made using private and public bicycles in cities where there exist bike sharing systems. PT: Public Transport; PM_2·5_: Particulate matter with diameter < 2.5 micrometers;

^a^ Year of data 2012 (except for Paris, 2011);

^b^ Suburban background monitor;

^c^ Expected mortality in both sexes;

^d^ Only shows the deaths per billion km travelled in public transport, and the risk does not include the 10 minutes walking;

* Data derived from secondary analysis of local data;

** Average from other cities (Table B in S1 File);

^+^ Average road fatalities between 1994–2012;

^++^ Estimate obtained from the trip distance and duration (Table B in S1 File).

### Road Traffic Fatality

We calculated the road traffic fatalities per billion kilometres travelled by mode of transport, based on fatal accidents occurring every year in each city related to the mode of transport and the kilometres travelled annually in each mode (Table B in S1 File). We then calculated the relative risk (RR) of all-cause mortality of road traffic fatality for each mode compared with the corresponding mode of transport and distance substituted. Two sensitivity analysis were performed for traffic fatalities, one base on a "safety in numbers" approach [16] and a second applying to all cities in scenario A Copenhagen’s death rate per kilometre travelled by bike and in scenario B Paris’ pedestrian death rate per kilometre travelled (Figs E-G in S1 File, Table P and Q in S1 File).

### Physical Activity

We estimated the duration of the trip, and the intensity of the activity using metabolic equivalent of task (MET) according to the mode of transport (Tables C-I in S1 File)[17]. We used a non-linear dose-response function for the RR of all-cause mortality for various levels of physical activity (in MET per hour per week) associated with each scenario and city to estimate the expected number of deaths [18]. We also performed a sensitivity analysis using a linear dose-response function between physical activity and all-cause mortality (Figs B-D in S1 File and Table O in S1 File) [13].

### Air Pollution Exposure

The air pollution assessment model considered the exposure to particulate matter with diameter less than 2.5μm (PM_2.5_), which has shown strong associations with all-cause mortality [19]. We compared exposure concentrations and inhaled dose for travel by car, bicycle, walk and public transport. To do so, we first obtained ratios of PM_2.5_ concentration levels in the car, bicycle, walking and public transport modes compared to a background site, from a previous study where we performed measurements in each mode of transport in Barcelona [20], which we then applied to each city’s average PM_2.5_ annual background concentration. We estimated yearly inhaled dose of PM_2.5_ accounting for mode-specific inhalation rates, exposures, and trip duration, as in de Nazelle et a l [20]. To estimate the RR of mortality associated with the change of air pollutant intake for travellers, we adjusted a concentration-based exposure-response function from the literature to an equivalent inhaled dose RR function [8]. We used a RR function for PM_2.5_ and all-cause mortality from a recent meta-analysis which included 11 international cohort studies (RR = 1.06 [1.04–1.08] for increment of 10μg/m^3^ of PM_2.5_) [19]. We also performed two sensitivity analysis, one using as a reference the RR function derived from a recent multicenter European study (RR = 1.07 [1.02–1.13] for increment of 5μg/m^3^ of PM_2.5_) [21], and a second assuming a fivefold toxicity of PM_2.5_ related to the emission source [22;23].

### Mortality Rates

We estimated the population attributable number of deaths for each scenario using the classic steps of HIA [24;25]. We applied the RR functions from the three domains to city specific all-cause mortality rates for the population between 16 and 64 years (Table 1).

### Carbon Dioxide Emissions

Carbon dioxide emissions (CO_2_) savings were estimated taking into account emission factors (kg of CO_2_/litre of fuel) and calibrated to the characteristics of vehicle fleets (share of vehicles using diesel or gasoline and engine efficiency) in each city (Table J in S1 File).

### Scenarios

The active transportation scenarios used in the six cities are presented in Table 2, and were compared to the business as usual (BAU) scenario for each city. The two scenarios included the following aims: A) attaining the levels of cycling in Copenhagen (35% of all trips by bicycle); B) attaining the levels of walking in Paris (50% of all trips walking). The distribution of the substitution was based on assumptions that were applied equally in the six cities (Table 2 and Table A in S1 File). The distance travelled in each scenario corresponds to distances made by each mode in the different cities The number of trips and distance by mode varies between cities (Tables 1 and 2) and are proportionally correlated with the health benefits and risk estimated.

**Table 2:** Active transportation scenarios.

Scenario	Description	Assumptions
**A**	Attaining the levels of cycling of the city of Copenhagen (35% of all trips in the city are made by bicycle)	50% of the trips coming from PT trips
		40% of the trips coming from Walk trips
		10% of the trips coming from Cars trips
**B**	Attaining the levels of walking of the city of Paris (50% of all trips in the city are made by walking)	75% of the trips coming from PT trips*
		1% of the trips coming from Bicycle trips*
		24% of the trips coming from Cars trips*

PT: Public Transport.

* Scenario B have different assumptions from scenario A based on the percentages of bicycle trips that could be substituted by walking trips in cities like Warsaw or Prague.

## Results

### Characteristics of the Six Cities

The number of inhabitants in the six cities ranged from 164,516 in Basel to 2,249,977 in Paris (Table 1). The percentage of trips by bicycle was low in Barcelona, Paris, Prague and Warsaw (≤3%) and higher in Basel and Copenhagen (≥15%). The number of fatal accidents per kilometre ranged from no deaths per billion kilometres travelled for public transport users in most cities to 122 deaths per billion kilometres travelled for pedestrians in Warsaw. Annual mean concentrations of PM_2.5_ ranged from 11μg/m^3^ in Copenhagen to 23.6μg/m^3^ in Warsaw. The scenarios and the re-distributions of the mode share are shown in Table 2 and Table A in S1 File.

### Attaining the Levels of Cycling of the City of Copenhagen (Scenario A)

**Fig 2:**
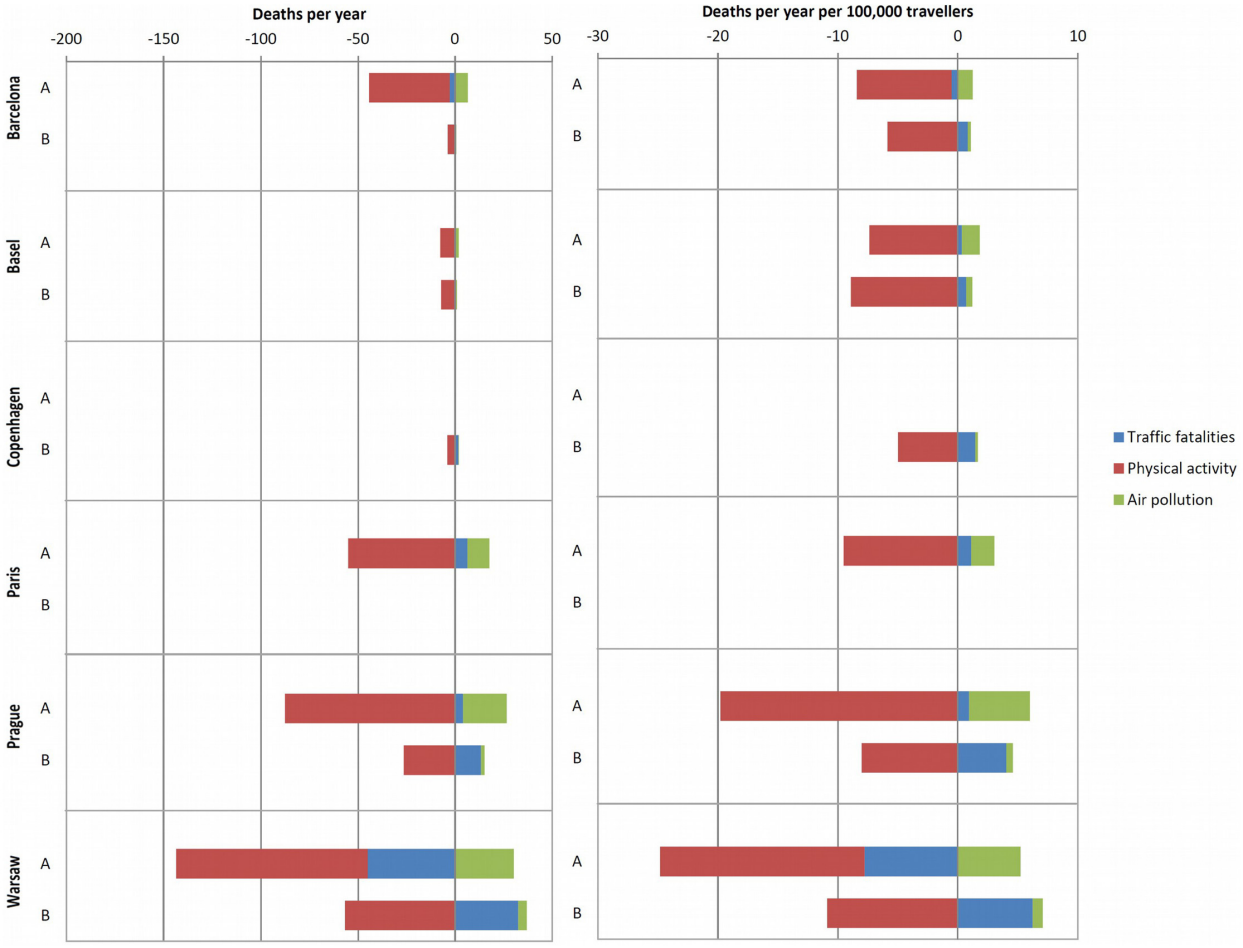
Number of deaths and Number of deaths adjusted by 100,000 travellers who shifted modes per year, by heath exposure. Scenarios. A: 35% of all trips by bicycle; B: 50% of all trips walking.

All five cities would have health benefit if 35% of trips were made by bicycle (not including Copenhagen, the reference city) (Table 3 and Fig 2). The estimated reduction in annual mortality would be 113.4 deaths in Warsaw, 61 in Prague, 37.8 in Barcelona, 37.4 in Paris and 5.7 in Basel.

**Table 3:** Number of deaths (95% confidence intervals) avoided or postponed per year and Number of deaths avoided or postponed per year per 100,000 travellers who shifted modes (95% confidence intervals).

Scenario	Deaths avoided per year (CI)	Barcelona	Basel	Copenhagen	Paris	Prague	Warsaw
**A**	35% of all trips by bicycles	-37.8 (-24, -56)	-5.7 (-3, -9)	-	-37.4 (-18, -64)	-61.0 (-29, -104)	-113.4 (-76, -163)
**B**	50% of all trips walking	-3.0 (-2, -4)	-6.2 (-4, -9)	-3.9 (-2, -6)	-	-11.3 (-3, -21)	-19.8 (-3, -42)
	Results by each 100,000 travellers who shifted modes* **(CI)**						
**A**	Cyclist increment	-7.1 (-4, -10)	-5.5 (-3, -9)	-	-6.5 (-3, -11)	-13.8 (-6, -23)	-19.6 (-13, -28)
**B**	Pedestrian increment	-4.7 (-3, -7)	-7.7 (-5, -11)	-3.1 (-1, -5)	-	-3.4 (-1, -6)	-3.8 (-1, -8)

CI: 95% confidence intervals;

* New cyclist or new pedestrians.

### Attaining the Levels of Walking of the City of Paris (Scenario B)

All five cities would have health benefits if 50% of trips were made walking (not including Paris for being the reference city) (Table 3 and Fig 2). There would be an estimated reduction of 19.8 deaths per year in Warsaw, 11.3 in Prague, 6.2 in Basel, 3.9 in Copenhagen and 3 in Barcelona.

### Carbon Dioxide Emissions

The maximum annual reduction in carbon dioxide emissions for the six cities would be for scenario A (35% of all trips by bicycles), with reductions ranging between 2,503 and 26,423 metric tons/year (Table M in S1 File).

## Discussion

This is the first study to quantify and compare health impacts of two active transportation targets in and between six European cities. These targets (35% cycling and 50% walking) come from real settings (Copenhagen and Paris), where active transportation policies have produced some of the greatest walking and cycling rates in Europe. The six European cities (Barcelona, Basel, Copenhagen, Paris, Prague and Warsaw) were chosen to obtain a diversity of active transport policies and characteristics in Europe (Table 1). For example, Copenhagen was included because of its high level of cycling while Barcelona, Prague, and Warsaw were included because of low levels of cycling. This study also incorporates more accurate methods to quantify health impacts (i.e. using a non-linear functions and local basal physical activity, to avoid over-estimation of the benefits of active transportation). Our results show that polices to promote active transportation are associated with health benefits in the six studied cities (Table 3). In absolute terms Warsaw would reap the most benefits, followed by Prague, Barcelona, Paris, then Basel, from the implementation of active transportation policies that favour the use of bicycles. Increasing the bicycling trips was also related with a higher reduction of CO_2_ emissions in the cities. The active transportation policies that promote walking in these cities would produce most benefits in Warsaw, followed by Prague, Basel, Copenhagen and Barcelona. Though results showed a small number of deaths avoided these results only show the mortality health impacts in travellers, and there are other co-benefits of such active transportation targets that could result in notable improvements in the quality of live in cities, including improvements in air quality, noise emissions, congestion, connectivity, accessibility, social interaction and cohesion, land use mixture, greenspaces, heat island effects, and others. Active transportation modes could also be perceived as pathways to improve the total urban environment.

Our results show that the implementation of active transportation targets aimed at improving the health of the population should take into account the characteristics of the city and may not be generalizable. When the results are adjusted by population size (100,000 travellers who shifted modes) to compare different cities (Table 3), in cities where pedestrian accident rates far exceeds those of cars, as is the case of Prague or Warsaw, a policy that only encourages walking without addressing road safety will not necessarily produce as high health benefits as in other cities. But Basel, which has the lowest accident rate for pedestrians of all six cities, is shown to benefit the most from the scenario (B) which stimulates walking trips. In the cycling scenario, Warsaw and Prague benefit the most, even though they have the highest accident risk for cycling of the six cities. This can be explained by the combination of the facts 1) they start from a very low baseline of cycling, hence reap more physical activity benefits from the scenario than other cities, and 2) 40% of the new cycle trips in the scenario come from walking trips, which have lower physical activity benefits but much higher fatal accident risk than cycling trips.

**Fig 3:**
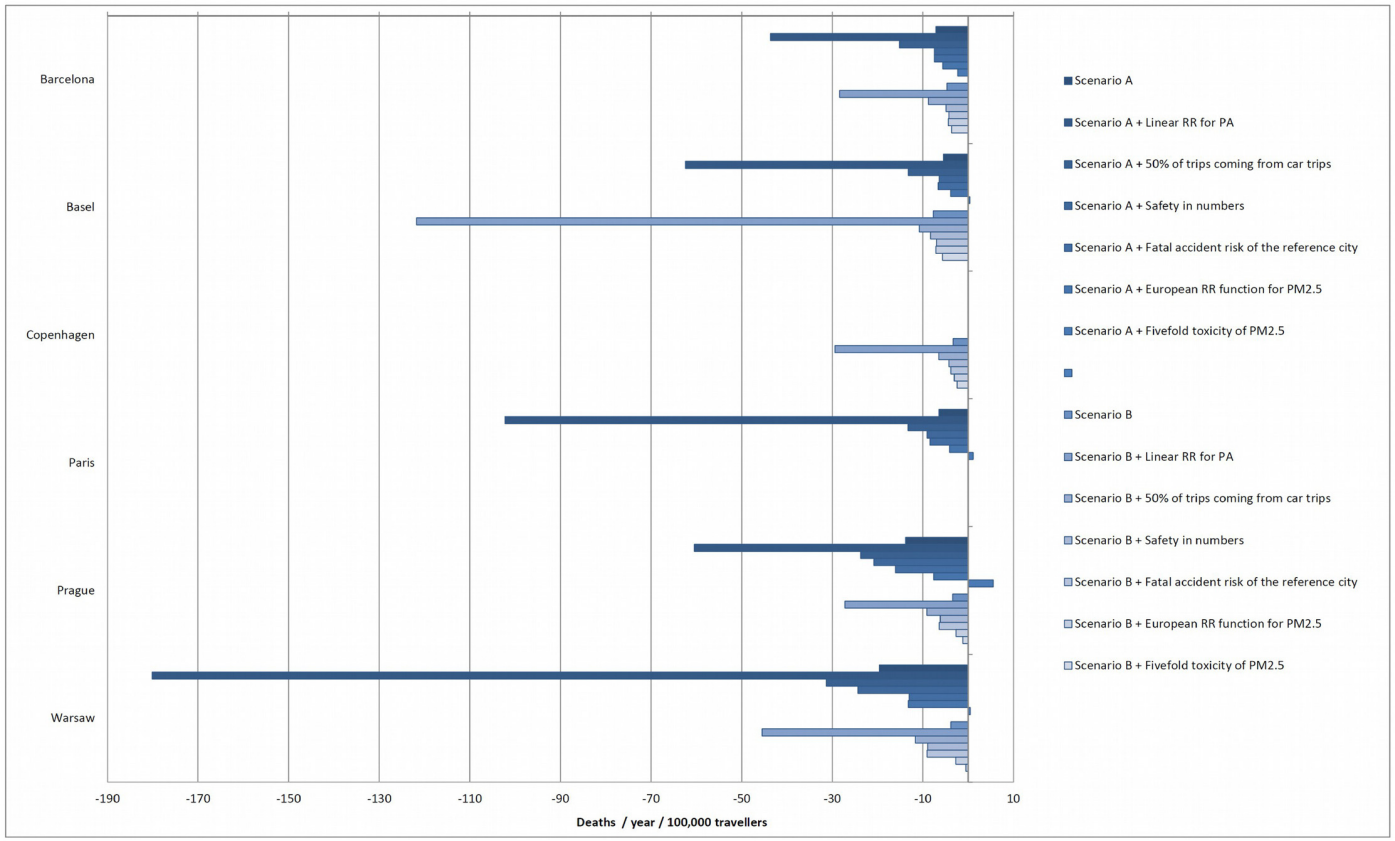
Sensitivity analysis. Number of deaths avoided or postponed per year by 100,000 travellers who shifted modes. A: Cyclist increment; B: Pedestrians increment. “Linear RR for PA”: Using a linear relative risk function for physical activity (for cycling or walking) as reported by kahlmeier S, et al, 2011; “50% of the trips coming from car trips”: Assume that half of the trips substituted in each scenario come from car trips; “Safety in numbers”: Assuming a fatal accident reduction associated with the increment of the number of pedestrians or cyclist; “Fatal accident risk of the reference city”: Assuming a fatal accident risk similar to the reference city for the scenarios A (fatal accident risk of cyclist in Copenhagen) and B (fatal accident risk of pedestrians in Paris); “European RR function for PM2.5”: Using a relative risk function of PM2.5 and all cause mortality reported in ESCAPE project (Beelen R, et al, 2014); “Fivefold toxicity of PM2.5”: Assuming a fivefold toxicity of PM2.5 from the traffic sources.

As in all risk assessments, our study was limited by the availability of data and the necessity to make assumptions to model likely scenarios. In particular, the policy directions depicted (walking/cycling increase) may only be interpreted within the strict limitation of the specific mode replacements which were assumed. However, by assuming in all cases that most mode shifts would occur from or towards public transport (e.g. from public transport to walk, Table 2 and Table A in S1 File), we provided rather conservative, but realistic scenarios. We also developed a sensitivity analysis assuming a less conservative scenario where 50% of the new cyclists or pedestrians come from car users (Fig 3, Table N in S1 File, and Fig A in S1 File). In this case we found that the health benefits doubled the estimates from the main results, showing the importance of the mode replacement assumptions and the relevance of promoting primarily a substitution from cars to active transportation.

Our study used a non-linear dose-response function (DRF) for physical activity and all-cause mortality and took into consideration the local basal levels of physical activity among travellers (Figs C-I in S1 File) [18;26;27]. This means that our model considers different benefits between people who are more sedentary [17]. This approach provides a more realistic estimate of the benefits of physical activity related to the increase in active transportation. The model assumes no substitution effect of leisure physical activity by active transportation, consistent with findings from recent studies based in Barcelona, England and United States [28–30]. Using a linear DRF and not accounting for basal levels of physical activity can overestimate the benefits of active transportation policies, as shown in previous studies [27]. We also performed a sensitivity analysis using linear DRFs for walking and cycling [13], and found that linear DRFs produced estimates of up to 10 times greater than those estimated with a non-linear DRF (Fig 3, Figs B-D in S1 File, and Table O in S1 File).

In the main analysis we assumed that traffic mortality rates remained the same after the policies were put in place. For that reason we performed a sensitivity analysis assuming the effect of what has been called “safety in numbers”, whereby increasing the number of trips made by cyclists or pedestrian, the individual risk of having a traffic accident will decrease [16]. We created an incidence rate ratio derived from the data from the six cities to estimate the effect of “safety in numbers” in our scenarios (Fig E in S1 File). We found greater health benefits, particularly in the case of cities like Prague or Warsaw where there are higher risks of traffic accidents for active transportation modes (Fig 3, Table P in S1 File and Fig F in S1 File). This increase in health benefits should be related to the reduction of cars on the streets, and potentially the improvement in safe infrastructure for cycling and walking, which were not formally assessed in this analysis, but may be partially accounted in our “safety in numbers” approach. To consider these factors we also performed a sensitivity analysis assuming for each scenario that the fatal accident risk of each city was the same as that of the reference city (fatal accident risk for cyclist in Copenhagen for scenario A and fatal accident risk for pedestrians in Paris for scenario B). Compared to the main analysis this sensitivity analysis yielded only a small increase in the health benefits for the cycling scenario across all the cities, and for the walking scenario in all but Barcelona and Basel (which currently have lower fatality rates for pedestrians than Paris) (Fig 3 and Table Q in S1 File and Fig G in S1 File).

Our approach for air pollution exposure focuses on the traveller who shifted the mode of transport, taking into consideration the inhalation rate and air pollution concentration in each mode of transport. Confirming other studies which have accounted for differences in inhalation of air pollution in various modes [7–9;14;31;32], we found that the health risks of exposure to air pollution (PM_2.5_) is much lower compared to the benefits of physical activity (Fig 2). Our main analysis of air pollution is based on the RR function between PM_2.5_ and all-cause mortality (1.06 per 10μg/m^3^ of PM_2.5_) derived from a recent meta-analysis [18]. We performed two sensitivity analyses regarding this RR function. The first assumed a RR function from a recent multicentre European study of long-term exposure to PM_2.5_ [21] which provides higher risk estimates (1.07 per 5μg/m^3^ of PM_2.5_) than the previous meta-analysis. This showed a slight decrement in the health benefits in all scenarios but maintained overall health benefits (Fig 3, Table R in S1 File and Fig H in S1 File).

The second sensitivity analysis was based on the assumption that traffic sources of PM2.5 can have a fivefold higher risk estimate [8;22] (Fig 3 and Table S in S1 File, and Fig I in S1 File). For cities like Prague, Warsaw and Paris, which have the highest concentrations of air pollution of the six cities, this fivefold toxicity factor produced small net harms rather than benefits. Our approach does not take into consideration the reduction of PM_2.5_ concentrations due to the reduction of car trips, which was considered marginal. This was shown in a study in Barcelona using an air pollution dispersion model, which did not identify a substantial reduction in PM_2.5_ concentration related to the reduction of car trips within the city (a reduction of 40% of car trips resulted in a 0.64% reduction in PM_2.5_ concentration) [7]. There are other factors that can change the health impacts of air pollution (e.g. cycling or walking infrastructure impacts on exposures). We did not consider these factors in our analysis because we expect they will produce only a minimal change in the results.

Previous health impact assessment studies have been performed in Barcelona, Copenhagen and Paris [7;9;14;31;32]. Contrary to these previous efforts, here we have used a non-linear approach to assess health impacts of physical activity. In addition, unlike the current study, the previous Copenhagen study did not include a pedestrian increase scenario. In all cases, the main finding of overall net health benefits of active travel policies remained the same.

Unlike studies which reported both morbidity and mortality outcomes [10;11;27;33], we only focused on mortality. We chose all-cause mortality as our main outcome to provide the most robust results possible, given the strongest evidence in the epidemiological literature for mortality. In addition, we did not stratify our results by age and gender. If the various stressors were to affect population with different age and gender groups differently [33], or if the mortality did not capture most of the adverse health effects, our approach might have created biased results. In this case we assume this bias to be small because we focus only on the adult working age population and because all-cause mortality is a cumulative outcome of multiple diseases, which also represents most of the burden of disease. Furthermore, it was shown previously that the estimation of all-cause mortality could be a reasonable indicator for morbidity impacts in HIA models for transport [31]. There is also some evidence of effect modification by sex, smoking status, obesity, and other health conditions which may vary by city, on outcomes of air pollution and physical activity, but to include these was beyond the scope of this paper. Our mortality estimates do not include a lag effect, but it could be expected that mortality estimates may only start to change after four to five years of meeting the active travel scenario targets.

Summarizing, this study highlights the importance of cities’ existing characteristics in determining health impacts of active transportation policies. Characteristics such as baseline physical activity levels in the population, traffic safety or air quality, can increase or decrease the benefits associated with active transportation policies. Such policies will produce higher health benefits when they focus on more sedentary population or more sedentary modes of transport (i.e. cars). The implementation of active transportation policies with an improvement in traffic safety (in particular for active transportation modes) will lead to greater net health benefits. And finally improving air quality beside the implementation of an active transportation policy will also reduce the risks for cyclist and pedestrians (as all other citizens) and increase the health benefits of the interventions. These characteristics should be taken into account by policy makers, stakeholders and risk assessors in devising policies.

## Conclusions

Active transportation policies can result in health benefits. To produce the greatest net health benefits, transport policies must first consider current traffic safety levels in the city and devise appropriate safety interventions for cyclists and pedestrians. The implementation of transport policies integrating a health perspective is needed in urban settings. To achieve this, a close and strong collaboration between health practitioners, transport specialists, and urban planners in cities is needed.

## Supporting Information

S1 File Supporting Information of the Health impacts of active transport in Europe. **Table A**. Description of the number of trips in each scenario by city and mode of transport (bold numbers refer to the objective of each scenario). **Table B**. Data sources of each city and area. **Table C.** Quartiles of basal level of physical activity reported in Barcelona travel survey. **Table D.** Percentages of basal levels of physical activity by sex and age reported in Switzerland. **Table E.** Percentages of basal levels of physical activity by sex and age reported in Denmark. **Table F.** Percentages of basal levels of physical activity by sex and age reported in France. **Table G.** Percentages of basal levels of physical activity by sex reported in Czech Republic. **Table H.** Percentages of basal levels of physical activity by age reported in Poland. **Table I.** Metabolic equivalent of task for each activity used for the analysis of air pollution and physical activity. **Table J.** Carbon dioxide emission factors and vehicle fleet description by city. **Table K.** Number of deaths per year estimated in each city by scenario and heath exposure. **Table L.** Number of deaths per year by 100,000 travellers estimated in each city by scenario and heath exposure. **Table M.** CO_2_ emissions (metric tons per year) avoided in each scenario and city. **Table N**. Number of deaths avoided or postponed per year per 100,000 travellers (95% confidence intervals) by scenario and city (related to physical activity, air pollution and road traffic fatalities), assuming a 50% of trips coming from car trips. **Fig A.** Number of deaths per year by 100,000 travellers by scenario, health exposure and city, assuming a 50% of trips coming from car trips. **Fig B.** Dose responses functions for physical activity and all-cause mortality. **Table O.** Number of deaths avoided or postponed per year per 100,000 travellers (95% confidence intervals) by scenario and city (related to physical activity, air pollution and road traffic fatalities), assuming a linear dose response function for walking and cycling and all-cause mortality. **Fig C.** Number of deaths per year by 100,000 travellers by scenario and each city (related to physical activity, air pollution and road traffic fatalities), comparing non-linear vs linear dose response function for physical activity and all-cause mortality. **Fig D.** Number of deaths per year by 100,000 travellers by scenario, health exposure and city, using a linear dose response function for physical activity and all-cause mortality. **Fig E.** Incidence Rate Ratio of fatal traffic accidents and number of cyclist and pedestrians used in the "safety in numbers" approach. **Table P.** Number of deaths avoided or postponed per year per 100,000 travellers (95% confidence intervals) by scenario and city (related to physical activity, air pollution and road traffic fatalities), using a quantitative approach of “safety in numbers”. **Fig F.** Number of deaths per year by 100,000 travellers by scenario, health exposure and city, using a quantitative approach of "safety in numbers". **Table Q.** Number of deaths avoided or postponed per year per 100,000 travellers (95% confidence intervals) by scenario and city (related to physical activity, air pollution and road traffic fatalities) applying to all the cities Copenhagen’s death rate per kilometre travelled by bike for scenario A and Paris’ pedestrian deaths rate per kilometre travelled for scenario B. **Fig G.** Number of deaths per year by 100,000 travellers by scenario, health exposure and city, applying to all the cities Copenhagen’s death rate per kilometre travelled by bike for scenario A and Paris’ pedestrian deaths rate per kilometre travelled for scenario B. **Table R.** Number of deaths avoided or postponed per year per 100,000 travellers (95% confidence intervals) by scenario and city (related to physical activity, air pollution and road traffic fatalities), assuming a dose response function of air pollution (PM2.5) and all-cause mortality (1.07 per 5 μg/m^3^) derived from the ESCAPE project (European Study of Cohorts for Air Pollution Effects). **Fig H.** Number of deaths per year by 100,000 travellers by scenario, health exposure and city, assuming a dose response function of air pollution (PM2.5) and all-cause mortality (1.07 per 5 μg/m^3^) derived from the ESCAPE project (European Study of Cohorts for Air Pollution Effects). **Table S.** Number of deaths avoided or postponed per year per 100,000 travellers (95% confidence intervals) by scenario and city (related to physical activity, air pollution and road traffic fatalities), assuming a 5 fold times more toxicity of air pollution. **Fig I.** Number of deaths per year by 100,000 travellers by scenario, health exposure and city, assuming a 5 fold times more toxicity of air pollution.
(PDF)
