# Supplementary material for: Health Impacts of Active Transportation in Europe
Source: PLoS One. 2016 Mar 1;11(3):e0149990. doi: 10.1371/journal.pone.0149990 (PMC4773008; doi:10.1371/journal.pone.0149990)
Supplement: S1 File — Table A. Description of the number of trips in each scenario by city and mode of transport (bold numbers refer to the objective of each scenario). Table B. Data sources of each city and area. Table C. Quartiles of basal level of physical activity reported in Barcelona travel survey. Table D. Percentages of basal levels of physical activity by sex and age reported in Switzerland. Table E. Percentages of basal levels of physical activity by sex and age reported in Denmark. Table F. Percentages of basal levels of physical activity by sex and age reported in France. Table G. Percentages of basal levels of physical activity by sex reported in Czech Republic. Table H. Percentages of basal levels of physical activity by age reported in Poland. Table I. Metabolic equivalent of task for each activity used for the analysis of air pollution and physical activity. Table J. Carbon dioxide emission factors and vehicle fleet description by city. Table K. Number of deaths per year estimated in each city by scenario and heath exposure. Table L. Number of deaths per year by 100,000 travellers estimated in each city by scenario and heath exposure. Table M. CO2 emissions (metric tons per year) avoided in each scenario and city. Table N. Number of deaths avoided or postponed per year per 100,000 travellers (95% confidence intervals) by scenario and city (related to physical activity, air pollution and road traffic fatalities), assuming a 50% of trips coming from car trips. Fig A. Number of deaths per year by 100,000 travellers by scenario, health exposure and city, assuming a 50% of trips coming from car trips. Fig B. Dose responses functions for physical activity and all-cause mortality. Table O. Number of deaths avoided or postponed per year per 100,000 travellers (95% confidence intervals) by scenario and city (related to physical activity, air pollution and road traffic fatalities), assuming a linear dose response function for walking and cycling and all-cause mortality. Fig C. Numb [file pone.0149990.s001.pdf]

Supporting Information

Health impacts of active transport in Europe.

Authors

David Rojas-Rueda, Audrey de Nazelle, Zorana Jovanovic Andersen, Charlotte Braun-Fahrländer, Jan Bruha, Hana Bruhova-Foltynova, Hélène Desqueyroux, Corinne Praznoczy, Martina S. Ragettli, Marko Tainio, Mark J Nieuwenhuijsen.

Centre for Research in Environmental Epidemiology, Municipal Institute of Medical Research, Universitat Pompeu Fabra, Departament de Ciències Experimentals i de la Salut and CIBER Epidemiología y Salud Pública, Barcelona, Spain (Rojas-Rueda D PhD, Prof Nieuwenhuijsen M PhD); Imperial College London, London, UK (de Nazelle A PhD);Center for Epidemiology and Screening, Department of Public Health, University of Copenhagen, Copenhagen, Denmark (Andersen ZJ PhD); Swiss Tropical and Public Health Institute, Basel, Switzerland (Prof Braun-Fahrländer C PhD, Ragettli M S PhD); The Kolin Institute of Technology, Prague, Czech Republic (Bruha J PhD, Bruhova-Foltynova H PhD); Agency for Environment and Energy Management, Paris, France (Desqueyroux H PhD); CEARC, OVSQ, Université de Versailles-Saint-Quentin-en-Yvelines, France (Praznoczy C PhD); UKCRC Centre for Diet and Activity Research (CEDAR), MRC Epidemiology Unit, University of Cambridge, Cambridge, UK (Tainio M PhD).

Table of contents

Section 1. Methods……………………………………………………………………………….…..4
1.1. Scenarios...4
Table 1. Description of the number of trips in each scenario by city and mode of transport (bold numbers refer to the objective of each scenario)………...4
1.2. Input data. 5
Table 2. Data sources of each city and area..5
1.3. Physical activity. 6
Table 3. Quartiles of basal level of physical activity reported in Barcelona travel survey..................6
Table 4. Percentages of basal levels of physical activity by sex and age reported in Switzerland.......6
Table 5. Percentages of basal levels of physical activity by sex and age reported in Denmark...........7
Table 6. Percentages of basal levels of physical activity by sex and age reported in France...............7
Table 7. Percentages of basal levels of physical activity by sex reported in Czech Republic..............8
Table 8. Percentages of basal levels of physical activity by age reported in Poland...........................8
Table 9. Metabolic equivalent of task for each activity used for the analysis of air pollution and physical activity..9
1.4. Public transport trips...10
1.5. Carbon dioxide...10
Table 10. Carbon dioxide emission factors and vehicle fleet description by city................................10

Section 2. Results..11
Table 11. Number of deaths per year estimated in each city by scenario and heath exposure...........11
Table 12. Number of deaths per year by 100,000 travellers estimated in each city by scenario and heath exposure. 12
Table 13. CO_2 emissions (metric tons per year) avoided in each scenario and city............................13

Section 3. Sensitivity analysis..14
3.1. Car trips substitution. 14
Table 14. Number of deaths avoided or postponed per year per 100,000 travellers (95% confidence intervals) by scenario and city (related to physical activity, air pollution and road traffic fatalities), assuming a 50% of trips coming from car trips...14
Fig 1. Number of deaths per year by 100,000 travellers by scenario, health exposure and city, assuming a 50% of trips coming from car trips...15

3.1. Physical activity..16
Fig 2. Dose responses functions for physical activity and all-cause mortality.....................................16
Table 15. Number of deaths avoided or postponed per year per 100,000 travellers (95% confidence intervals) by scenario and city (related to physical activity, air pollution and road traffic fatalities), assuming a linear dose response function for walking and cycling and all-cause mortality...17
Fig 3. Number of deaths per year by 100,000 travellers by scenario and each city

(related to physical activity, air pollution and road traffic fatalities), comparing non-linear vs linear dose response function for physical activity and all-cause mortality....18
Fig 4. Number of deaths per year by 100,000 travellers by scenario, health exposure and city, using a linear dose response function for physical activity and all-cause mortality....19

3.2. Road traffic fatality....20
Fig 5. Incidence Rate Ratio of fatal traffic accidents and number of cyclist and pedestrians used in the "safety in numbers" approach....20
Table 16. Number of deaths avoided or postponed per year per 100,000 travellers (95% confidence intervals) by scenario and city (related to physical activity, air pollution and road traffic fatalities), using a quantitative approach of “safety in numbers”....21
Fig 6. Number of deaths per year by 100,000 travellers by scenario, health exposure and city, using a quantitative approach of "safety in numbers"....22
Table 17. Number of deaths avoided or postponed per year per 100,000 travellers (95% confidence intervals) by scenario and city (related to physical activity, air pollution and road traffic fatalities) applying to all the cities Copenhagen’s death rate per kilometre travelled by bike for scenario A and Paris’ pedestrian deaths rate per kilometre travelled for scenario B....23
Fig 7. Number of deaths per year by 100,000 travellers by scenario, health exposure and city, applying to all the cities Copenhagen’s death rate per kilometre travelled by bike for scenario A and Paris’ pedestrian deaths rate per kilometre travelled for scenario B....24

3.3. Air pollution....25
Table 18. Number of deaths avoided or postponed per year per 100,000 travellers (95% confidence intervals) by scenario and city (related to physical activity, air pollution and road traffic fatalities), assuming a dose response function of air pollution (PM2.5) and all-cause mortality (1.07 per 5 $\mu g/m^3$) derived from the ESCAPE project (European Study of Cohorts for Air Pollution Effects)....25
Fig 8. Number of deaths per year by 100,000 travellers by scenario, health exposure and city, assuming a dose response function of air pollution (PM2.5) and all-cause mortality (1.07 per 5 $\mu g/m^3$) derived from the ESCAPE project (European Study of Cohorts for Air Pollution Effects)....26
Table 19. Number of deaths avoided or postponed per year per 100,000 travellers (95% confidence intervals) by scenario and city (related to physical activity, air pollution and road traffic fatalities), assuming a 5 fold times more toxicity of air pollution....27
Fig 9. Number of deaths per year by 100,000 travellers by scenario, health exposure and city, assuming a 5 fold times more toxicity of air pollution....28

References....29

Section 1. Methods.

1.1. Scenarios.

Table 1. Description of the number of trips in each scenario by city and mode of transport (bold numbers refer to the objective of each scenario).

	Scenario	Walking	Bicycling	PT	Car
Barcelona	BAU	2.302.569	109.282	1.484.788	457.095
	**A**	1.646.376	**1.749.763**	664.547	293.047
	**B**	**2.499.662**	107.311	1.336.968	409.793
Basel	BAU	608.808	265.186	443.900	429.320
	**A**	468.036	**617.117**	267.934	394.127
	**B**	**881.596**	262.458	239.309	363.851
Copenhagen	BAU	520.615	492.805	303.333	491.576
	**A**	NA	NA	NA	NA
	**B**	**904.165**	488.970	15.671	399.524
Paris	BAU	2.819.239	162.147	2.027.880	731.482
	**A**	2.033.343	**2.126.886**	1.045.510	535.008
	**B**	NA	NA	NA	NA
Prague	BAU	888.383	9.737	1.860.517	932.643
	**A**	375.202	**1.292.691**	1.219.040	804.348
	**B**	**1.846.701**	154	1.141.779	702.647
Warsaw	BAU	997.820	54.818	2.520.225	1.278.847
	**A**	303.533	**1.790.536**	1.652.366	1.105.275
	**B**	**2.557.909**	39.217	1.350.158	904.426

PT: Public transport; BAU Business as usual; NA: Not applicable; Scenario A: 35% of all trips by bicycle; Scenario B: 50% of all trips walking.

Scenarios were built based on assumptions about the modal distribution and shifting between modes of transport, focused on increasing active transport assumed that most of the trips would be substitutes from public transport.

1.2. Input data.

All the transport data included in the analysis come from official records or transport surveys from each city. This data may have been collected using different methods but are the best available data. However we acknowledge that there is some uncertainty in the data and that the data may have been more reliable had it been collected using the same methodology, with preferably objective assessment methods such as automatic counters and automatic tracking of individuals

Table 2. Data sources of each city and area.

	Barcelona	Basel	Copenhagen	Paris	Prague	Warsaw
**Transport data**	Travel survey	Travel survey	Travel survey	Travel survey	Travel survey	Travel survey
**Air quality data**	Air quality monitors	Air quality monitors	Air quality monitors	Air quality monitors	Air quality monitors	Air quality monitors
**Health data**	Health records	Health records	Health records	Health records	Health records	Health records
**Demographic data**	City records	City records	City records	City records	City records	City records
**Traffic safety data**	Police reports	Police reports	Police reports	Police reports	Police reports	Police reports

Assumptions: Where input variables in Table-2 of the main text were not directly available from sources consulted in each city, either 1) they were derived from secondary analysis of primary data available in the same city (see below for detailed explanation), or 2) when data was not available for secondary analysis either, the average value of the other cities was used as input,.

Average distance travelled per mode: Obtained directly from the city records. In cases where the city records did not report this data, it was estimated based on the average trip duration and average speed reported by the city. Cities where this was applied: Barcelona, Copenhagen and Warsaw.

Average speed: Obtained directly from the city records. In cases where the city records did not report this data, it was estimated based on the average distance travelled and average trip duration reported by the city. City where this was applied: Basel and Warsaw.

Road traffic fatalities per year: This data was obtained for all ages combined, directly from city records. To adjusted for the 16 to 64 years old age group, a 0.77 ratio was applied to overall traffic mortality, based on a report from traffic fatalities in Europe which showed that the 77% of the traffic fatalities were suffered by this ages group (1)**.**

Deaths per billion kilometre travelled: Calculated in each city based on road traffic fatalities reported per year and the distance travelled per year by mode in each city.

Concentration of $PM_{2.5}$: The city annual average was obtained from the city records. The concentration in each mode of transport were estimated based on the study performed in Barcelona (2) and adjusted by the annual average concentration of each city.

1.3. Physical activity.

The health impacts of increased physical activity by active transport taking into account baseline levels of physical activity (METs/H/w) in each city by age group and sex, which we obtained from local and national records and surveys. The baseline levels of physical activity used by the model in each city are presented in the tables 3 to 8 in METs/h/w.

Table 3. Quartiles of basal level of physical activity reported in Barcelona travel survey.

Quartile	METs/h/w
Q1	0·4
Q2	8·5
Q3	22·5
Q4	42·4

METs/h/w : Metabolic equivalent of task per hour per week (e.g. 8·75 METs/h/w = 30 min of walking 7 days per week; 22·05 METs/h/w = 27 min of jogging 7 days per week; 40·83 METs/h/w = 50 min of jogging 7 days per week).

Table 4. Percentages of basal levels of physical activity by sex and age reported in Switzerland.

		Man				Woman			
**Physical activity levels**	**METs/H/w**	15-34 years	35-49 years	50-64 years	=> 65 years	15-34 years	35-49 years	50-64 years	=> 65 years
**Trained**	45	43	27	23	23	30	23	23	13
**Regular active**	37·5	37	43	50	50	43	43	43	47
**Partially active**	15	13	20	17	13	20	20	20	17
**Inactive**	0	7	10	10	13	7	13	13	23

METs/h/w : Metabolic equivalent of task per hour per week (e.g. 8·75 METs/h/w = 30 min of walking 7 days per week; 22·05 METs/h/w = 27 min of jogging 7 days per week; 40·83 METs/h/w = 50 min of jogging 7 days per week).

Table 5. Percentages of basal levels of physical activity by sex and age reported in Denmark.

		Man					Woman				
**Physical activity levels**	**METs/H/w**	15-24 years	25-44 years	45-64 years	65-79 years	=> 80 years	15-24 years	25-44 years	45-64 years	65-79 years	=> 80 years
**Competitive sports**	30	26	7	2	1	0	9	3	1	0	0
**Heavy physical exercise**	24	30	34	25	18	6	29	24	16	10	3
**Light physical exercise**	12	32	45	60	65	56	51	63	72	72	48
**Sedentary**	0	12	13	11	14	34	10	10	10	16	45

METs/h/w : Metabolic equivalent of task per hour per week (e.g. 8·75 METs/h/w = 30 min of walking 7 days per week; 22·05 METs/h/w = 27 min of jogging 7 days per week; 40·83 METs/h/w = 50 min of jogging 7 days per week).

Table 6. Percentages of basal levels of physical activity by sex and age reported in France.

		Man						Woman					
**Physical activity levels**	**METs/H/w**	15-25 years	26-34 years	35-44 years	45-54 years	55-64 years	65-75 years	15-25 years	26-34 years	35-44 years	45-54 years	55-64 years	65-75 years
**High**	25	68	55	48	43	45	43	32	32	36	30	33	39
**Middle**	17·5	16	17	14	19	24	27	33	27	26	26	27	32
**Limited**	5	14	26	36	37	29	28	34	39	36	43	38	28

METs/h/w : Metabolic equivalent of task per hour per week (e.g. 8·75 METs/h/w = 30 min of walking 7 days per week; 22·05 METs/h/w = 27 min of jogging 7 days per week; 40·83 METs/h/w = 50 min of jogging 7 days per week).

Table 7. Percentages of basal levels of physical activity by sex reported in Czech Republic.

Physical activity levels	METs/H/w	Man	Woman
**High**	40	53	23·7
**Middle**	20	22·6	42·7
**Sedentary**	0	25	33

METs/h/w : Metabolic equivalent of task per hour per week (e.g. 8·75 METs/h/w = 30 min of walking 7 days per week; 22·05 METs/h/w = 27 min of jogging 7 days per week; 40·83 METs/h/w = 50 min of jogging 7 days per week).

Table 8. Percentages of basal levels of physical activity by age reported in Poland.

Physical activity levels	METs/H/w	15-19 years	20-29 years	30-39 years	40-49 years	50-59 years	60-69 years	70-79 years	=>80 years
**High**	58·2	28	36	39	38	27	13	5	1
**Middle**	38	64	68	72	74	70	64	53	37
**Sedentary**	0	81	77	76	75	77	80	73	60

METs/h/w : Metabolic equivalent of task per hour per week (e.g. 8·75 METs/h/w = 30 min of walking 7 days per week; 22·05 METs/h/w = 27 min of jogging 7 days per week; 40·83 METs/h/w = 50 min of jogging 7 days per week).

Table 9. Metabolic equivalent of task for each activity used for the analysis of air pollution and physical activity.(3)

Activity	METs/h/w
Sleep	0·9
Rest	1·0
Riding in a bus	1·0*
Driving an automobile	2·0
Walking	2·5
Bicycling 10-11·9 mph, light effort	6·8

METs/h/w : Metabolic equivalent of task per hour per week.
*This was applied to all the public transport modes.

1.4. Public transport trips

Public transport trips included metro, train, bus, and tram trips, depending on the city.

Was assumed that public transport trips involved 10 minutes of walking and was included in the model the benefits of physical activity, the risk of suffering a road traffic fatality as a pedestrian and the inhalation of air pollution during the 10 minutes walking in the risk associated with public transport.

1.5. Carbon dioxide.

Table 10. Carbon dioxide emission factors and vehicle fleet description by city.

		Barcelona	Basel	Copenhagen	Paris	Prague	Warsaw
Percentage of cars in the city per type of fuel (%)	Gasoline	56·0	67·6	60·1	60·1	56·7	60·1
	Diesel	44·0	30·5	39·3	39·3	43·3	39·3
Efficiency of cars fleet in the city (L/100km)	Gasoline	9·0	5·2	7·1	7·1	7·1	7·1
	Diesel	7·0	3·9	5·5	5·5	5·7	5·5
CO_2 released (kg/L)	Gasoline	2·4	2·4	2·4	2·4	2·4	2·4
	Diesel	2·6	2·6	2·6	2·6	2·6	2·6

Data comes from the latest data reported available from each city. CO_2: Carbon dioxide.

Section 2. Results.

Table 11. Number of deaths per year estimated in each city by scenario and heath exposure.

Scenario	Road traffic fatalities	Physical activity	Air pollution
	**Barcelona**		
**A**	-2·73	-41·61	6·54
**B**	0·52	-3·71	0·17
	**Basel**		
**A**	0·34	-7·62	1·54
**B**	0·56	-7·17	0·40
	**Copenhagen**		
**A**	-	-	-
**B**	1·74	-3·97	0·25
	**Paris**		
**A**	6·41	-55·10	11·22
**B**	-	-	-
	**Prague**		
**A**	4·09	-87·60	22·47
**B**	13·34	-26·49	1·85
	**Warsaw**		
**A**	-44·98	-98·67	30·26
**B**	32·50	-56·74	4·36

Scenario A: 35% of all trips by bicycle; Scenario B: 50% of all trips walking. Negative numbers (-) mean avoided deaths; Positive numbers mean increased deaths.

Table 12. Number of deaths per year by 100,000 travellers estimated in each city by scenario and heath exposure.

Scenario	Road traffic fatalities	Physical activity	Air pollution
**Barcelona**			
**A**	-0·52	-7·89	1·24
**B**	0·83	-5·86	0·27
**Basel**			
**A**	0·33	-7·36	1·50
**B**	0·70	-8·93	0·51
**Copenhagen**			
**A**	-	-	-
**B**	1·46	-4·98	0·21
**Paris**			
**A**	1·11	-9·53	1·94
**B**	-	-	-
**Prague**			
**A**	0·93	-19·80	5·08
**B**	4·04	-8·01	0·56
**Warsaw**			
**A**	-7·77	-17·05	5·23
**B**	6·25	-10·91	0·84

Scenario A: 35% of all trips by bicycle; Scenario B: 50% of all trips walking. Negative numbers (-) mean avoided deaths; Positive numbers mean increased deaths.

Table 13. CO_2 emissions (metric tons per year) avoided in each scenario and city

**Scenario**		Barcelona	Basel	Copenhagen	Paris	Prague	Warsaw
**A**	35% of all trips by bicycles	22,957	2,503	-	19,923	22,819	26,423
**B**	50% of all trips walking	1,139	2,088	2,745	-	8,320	11,611

Section 3. Sensitivity analysis.

3.1. Car trips substitution.

Table 14. Number of deaths avoided or postponed **per year per 100,000 travellers (95% confidence intervals) by scenario and city (related to physical activity, air pollution and road traffic fatalities), assuming a 50% of trips coming from car trips.**

Scenario	Barcelona	Basel	Copenhagen	Paris	Prague	Warsaw
**Main result (CI)**						
**A**	-7·1<br>(-4, -10)	-5·5<br>(-3, -9)	-	-6·5<br>(-3, -11)	-13·8<br>(-6, -23)	-19·6<br>(-13, -28)
**B**	-4·7<br>(-3, -7)	-7·7<br>(-5, -11)	-3·1<br>(-1, -5)	-	-3·4<br>(-1, -6)	-3·8<br>(-1, -8)
**Sensitivity analysis , applying 50% of car trips substitution by bicycling or walking (CI)**						
**A**	-15·2<br>(-10, -22)	-13·2<br>(-8, -20)	-	-13·2<br>(-8, -21)	-23·7<br>(-13, -39)	-31·4<br>(-20, -47)
**B**	-8·8<br>(-6, -12)	-10·8<br>(-7, -16)	-6·5<br>(-4, -10)	-	-9·1<br>(-5, -14)	-11·6<br>(-7, -19)

Scenario A: 35% of all trips by bicycle; Scenario B: 50% of all trips walking. Negative numbers (-) mean avoided deaths; Positive numbers mean increased deaths. CI: Confidence intervals.

Fig 1. Number of deaths per year by 100,000 travellers by scenario, health exposure and city, assuming a 50% of trips coming from car trips.

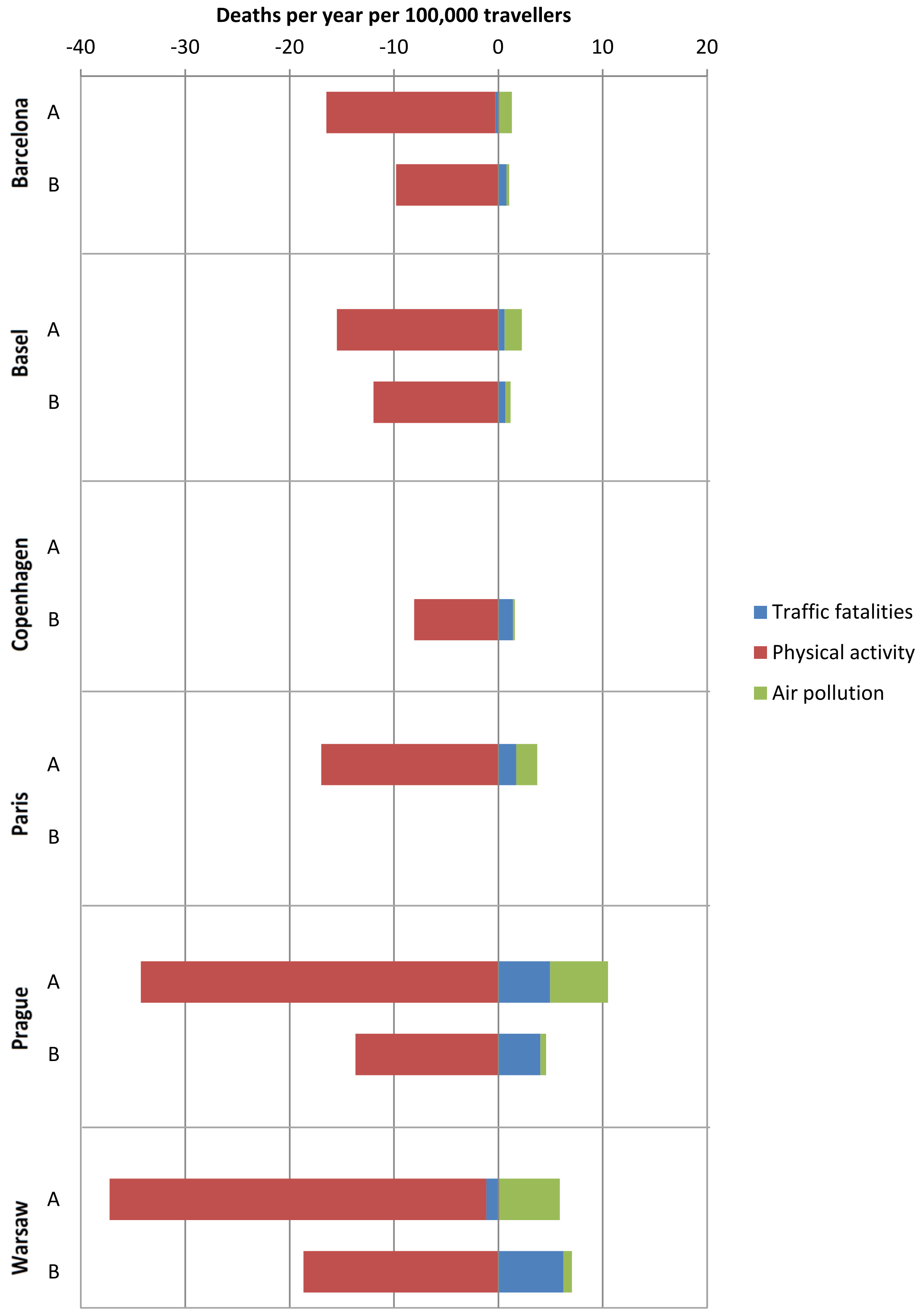

Scenario A: 35% of all trips by bicycle; Scenario B: 50% of all trips walking.

3.2. Physical activity.

Fig 2. Dose response functions for physical activity and all-cause mortality.

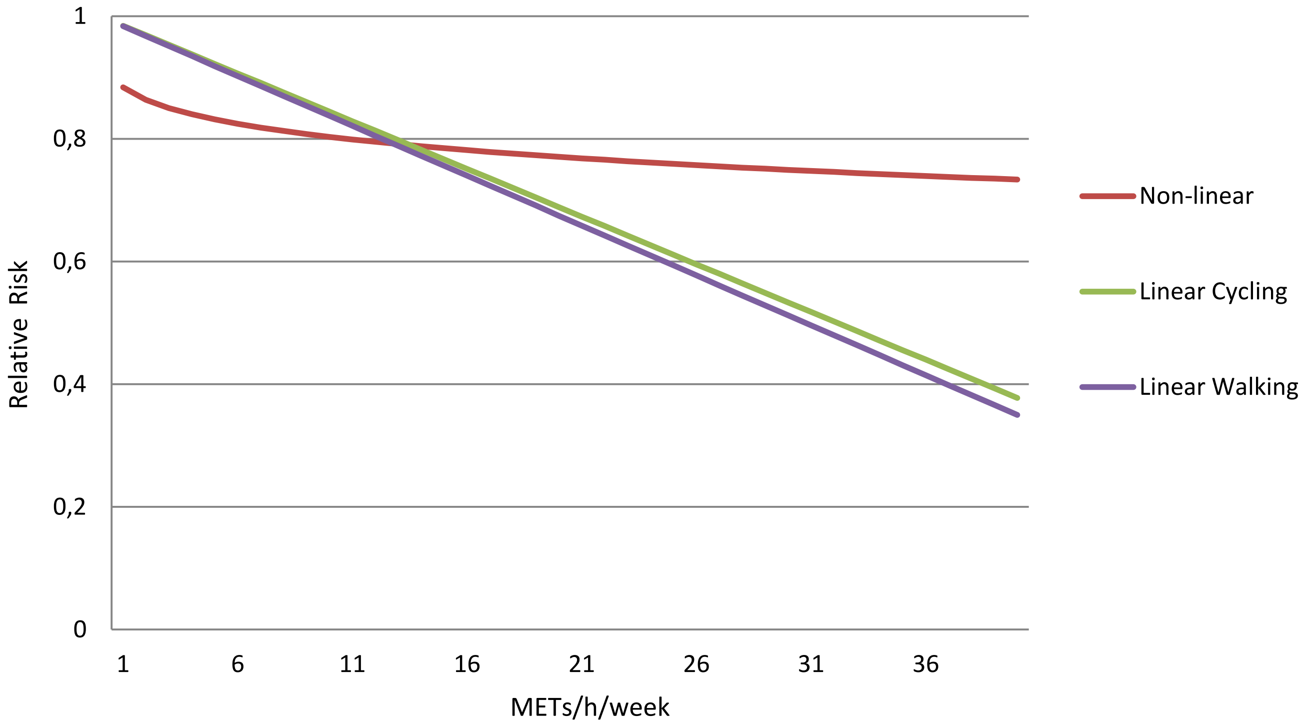

METs/h/w : Metabolic equivalent of task per hour per week.; Non-linear dose response function from Woodcock J et al, 2010(4); Linear Cycling and Linear Walking from Kahlmeier S, et al, 2011(5).

Table 15. Number of deaths avoided or postponed **per year per 100,000 travellers (95% confidence intervals) by scenario and city (related to physical activity, air pollution and road traffic fatalities), assuming a linear dose response function for walking and cycling and all-cause mortality.**

Scenario	Barcelona	Basel	Copenhagen	Paris	Prague	Warsaw
**Main result (CI)**						
**A**	-7·1<br>(-4, -10)	-5·5<br>(-3, -9)	-	-6·5<br>(-3, -11)	-13·8<br>(-6, -23)	-19·6<br>(-13, -28)
**B**	-4·7<br>(-3, -7)	-7·7<br>(-5, -11)	-3·1<br>(-1, -5)	-	-3·4<br>(-1, -6)	-3·8<br>(-1, -8)
**Sensitivity analysis , applying linear dose response function for physical activity (CI)**						
**A**	-43·6<br>(-26, -78)	-62·4<br>(-28, -93)	-	-102·2<br>(-34, -124)	-60·4<br>(-56, -112)	-180·1<br>(-74, -225)
**B**	-28·3<br>(-1, -62)	-121·7<br>(-4, -166)	-29·4<br>(-1, -65)	-	-27·2<br>(2, -73)	-45·4<br>(3, -153)

Scenario A: 35% of all trips by bicycle; Scenario B: 50% of all trips walking. RR Linear function for cycling and walking from Kahlmeier S, et al, 2011(5). Negative numbers (-) mean avoided deaths; Positive numbers mean increased deaths. Negative numbers (-) mean avoided deaths; Positive numbers mean increased deaths. CI: Confidence intervals.

Fig 3. Number of deaths per year by 100,000 travellers estimated for each scenario and each city (related to physical activity, air pollution and road traffic fatalities), comparing non-linear vs linear dose response function for physical activity and all-cause mortality.

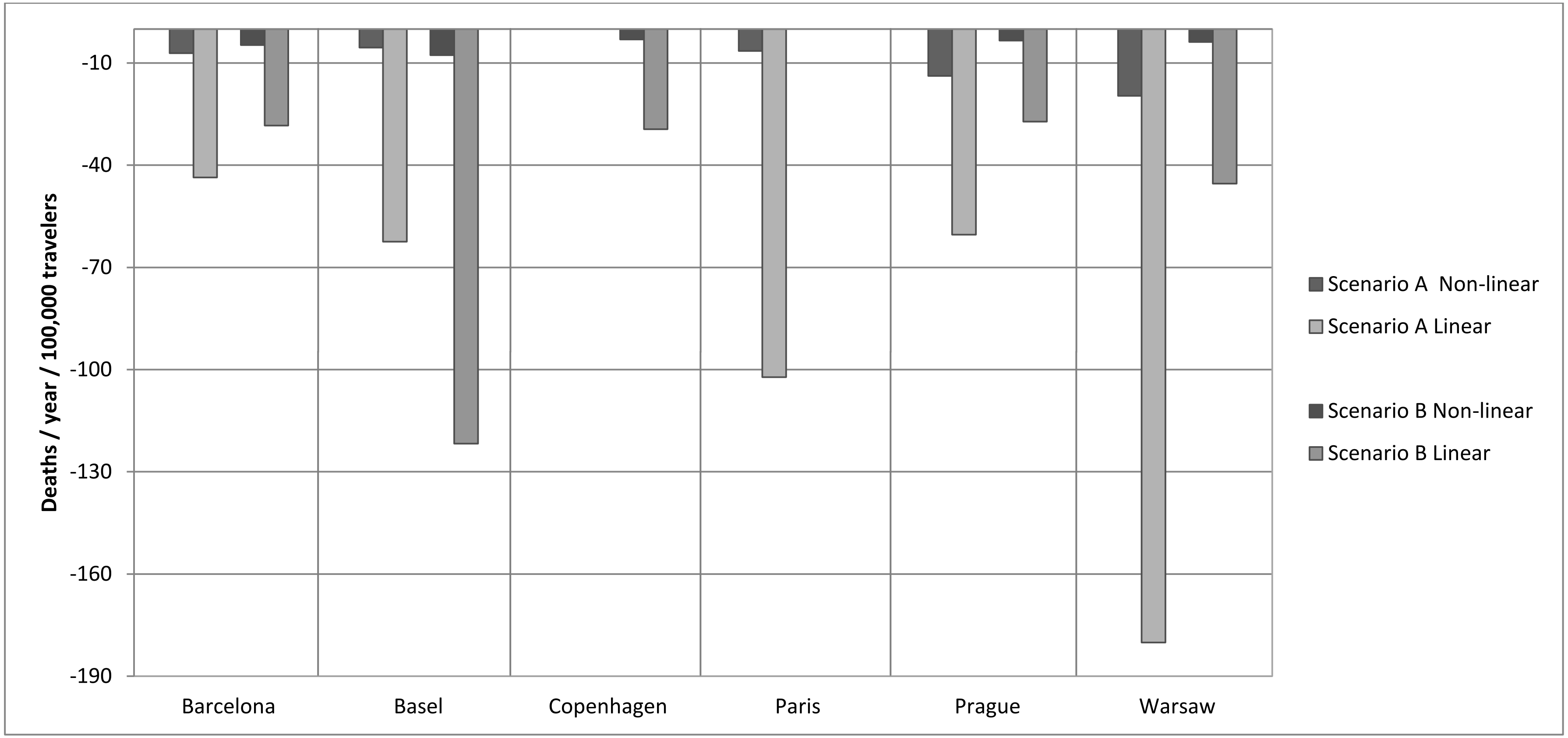

Scenario A: 35% of all trips by bicycle; Scenario B: 50% of all trips walking. Non-linear dose response function from Woodcock J et al, 2010(4); Linear Cycling and Linear Walking from Kahlmeier S, et al, 2011(5).

Fig 4. Number of deaths per year by 100,000 travellers by scenario, health exposure and city, using a linear dose response function for physical activity and all-cause mortality.

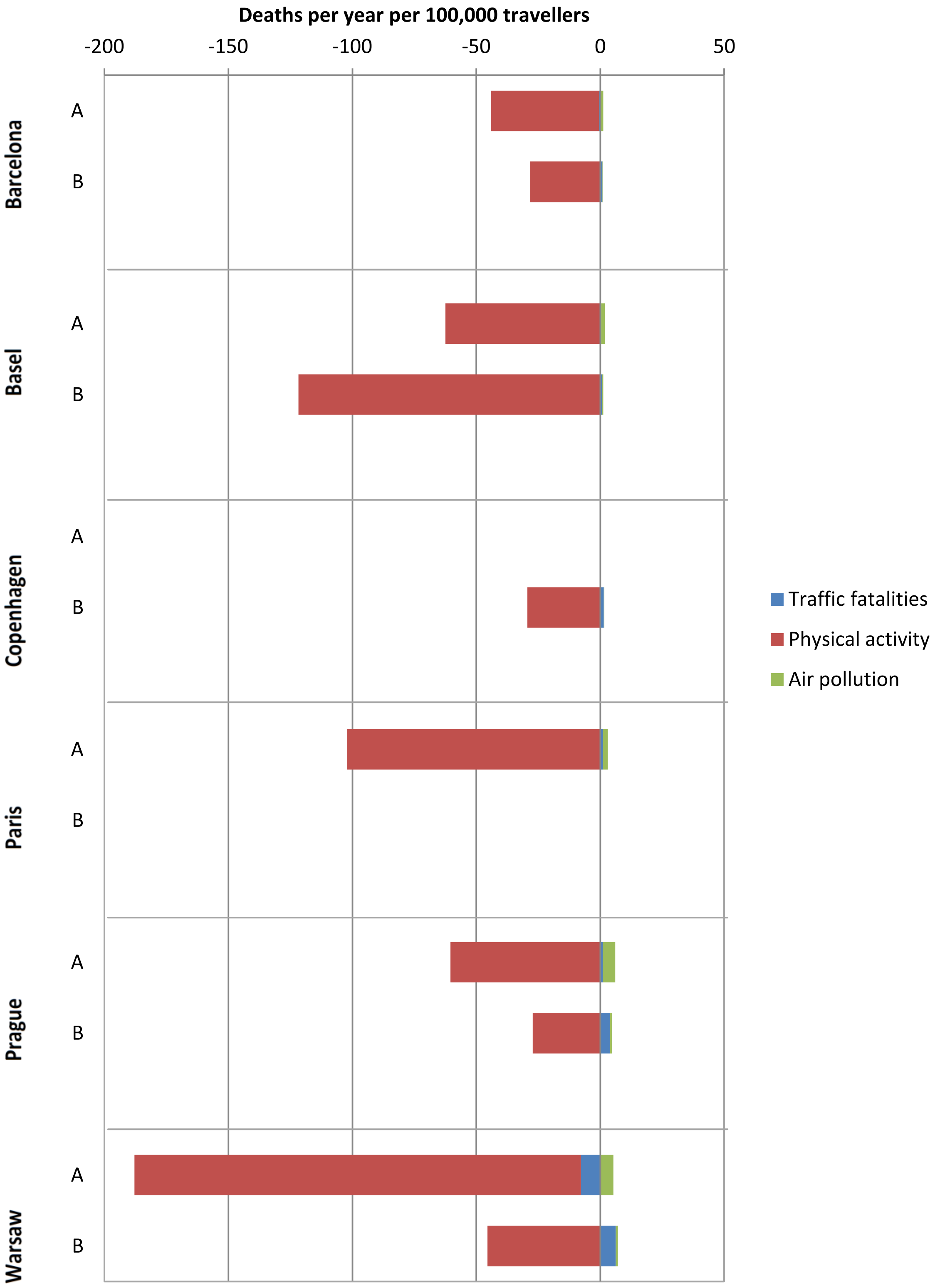

Scenario A: 35% of all trips by bicycle; Scenario B: 50% of all trips walking.

3.3. Road traffic fatality.

Fig 5. Incidence Rate Ratio of fatal traffic accidents and number of cyclist and pedestrians used in the "safety in numbers" approach.

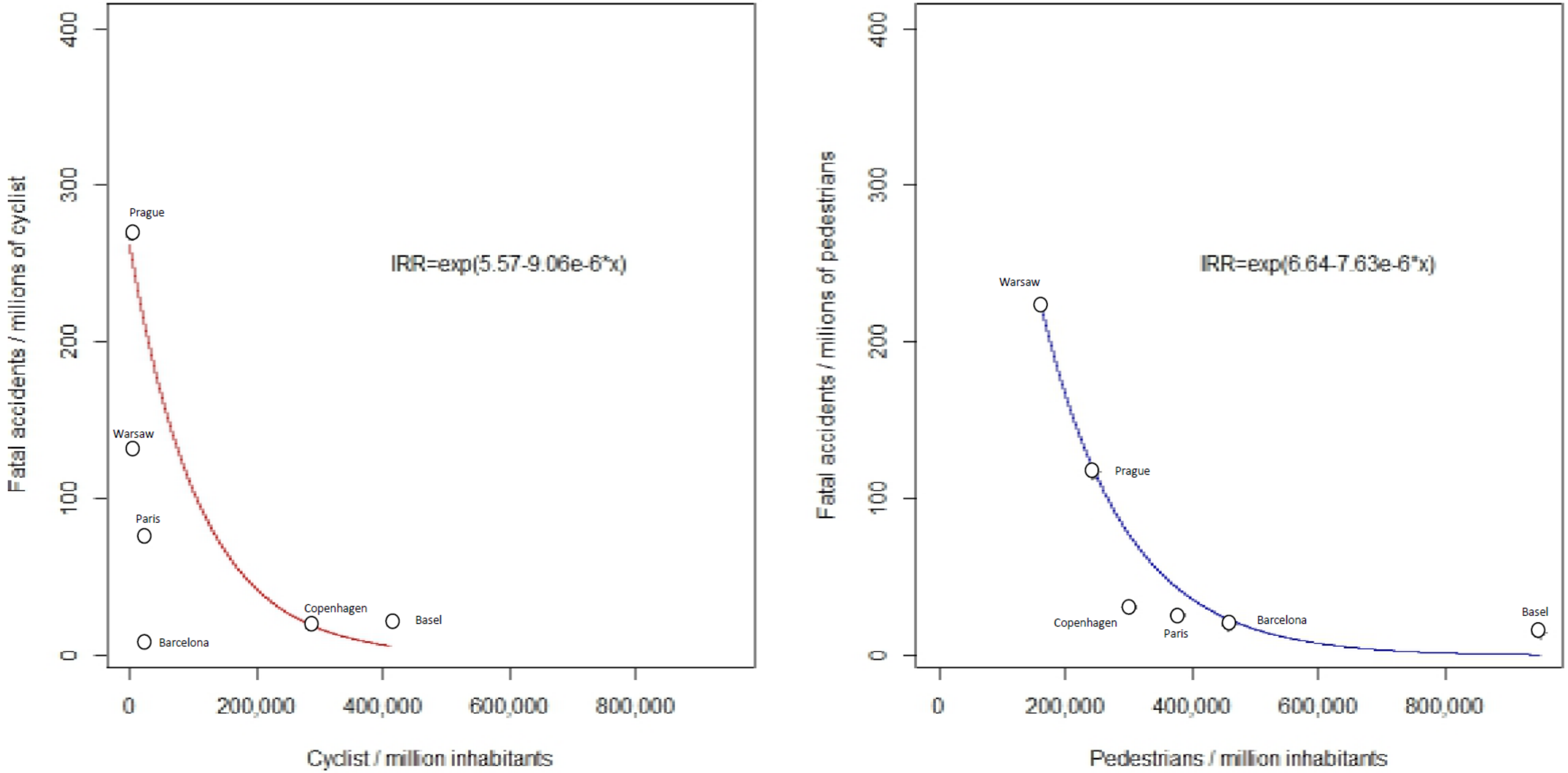

This graph shows a reduction in the fatal accidents per million of travellers (cyclist or pedestrians) when the number of travellers (cyclist or pedestrians) increases in the population. Base on these data from the six cities, a incidence rate ratio (IRR) of fatal accidents was estimated for cyclist or pedestrians. This IRR (for cyclist or pedestrians) was used to quantify the reduction of fatal accidents in the different cities according with the expected increment of travellers (cyclist or pedestrians) in each scenario.

Table 16. Number of deaths avoided or postponed **per year per 100,000 travellers (95% confidence intervals) by scenario and city (related to physical activity, air pollution and road traffic fatalities) using a quantitative approach of "safety in numbers".**

Scenario	Barcelona	Basel	Copenhagen	Paris	Prague	Warsaw
**Main result (CI)**						
**A**	-7·1 (-4, -10)	-5·5 (-3, -9)	-	-6·5 (-3, -11)	-13·8 (-6, -23)	-19·6 (-13, -28)
**B**	-4·7 (-3, -7)	-7·7 (-5, -11)	-3·1 (-1, -5)	-	-3·4 (-1, -6)	-3·8 (-1, -8)
**Sensitivity analysis, applying "safety in numbers" approach (CI)**						
**A**	-7·4 (-4, -11)	-6·3 (-3, -9)	-	-8·1 (-4, -12)	-20·8 (-13, -30)	-24·3 (-18, -33)
**B**	-4·9 (-3, -7)	-8·3 (-5, -12)	-4·2 (-2, -6)	-	-6·1 (-3, -9)	-8·9 (-5, -13)

Scenario A: 35% of all trips by bicycle; Scenario B: 50% of all trips walking. The "safety in numbers" approach, was based on the incidence rate ratio of fatal traffic accidents (in cyclists or pedestrians) estimated by the six cities data. Negative numbers (-) mean avoided deaths; Positive numbers mean increased deaths. CI: Confidence intervals.

Fig 6. Number of deaths per year by 100,000 travellers by scenario, health exposure and city, using a quantitative approach of "safety in numbers".

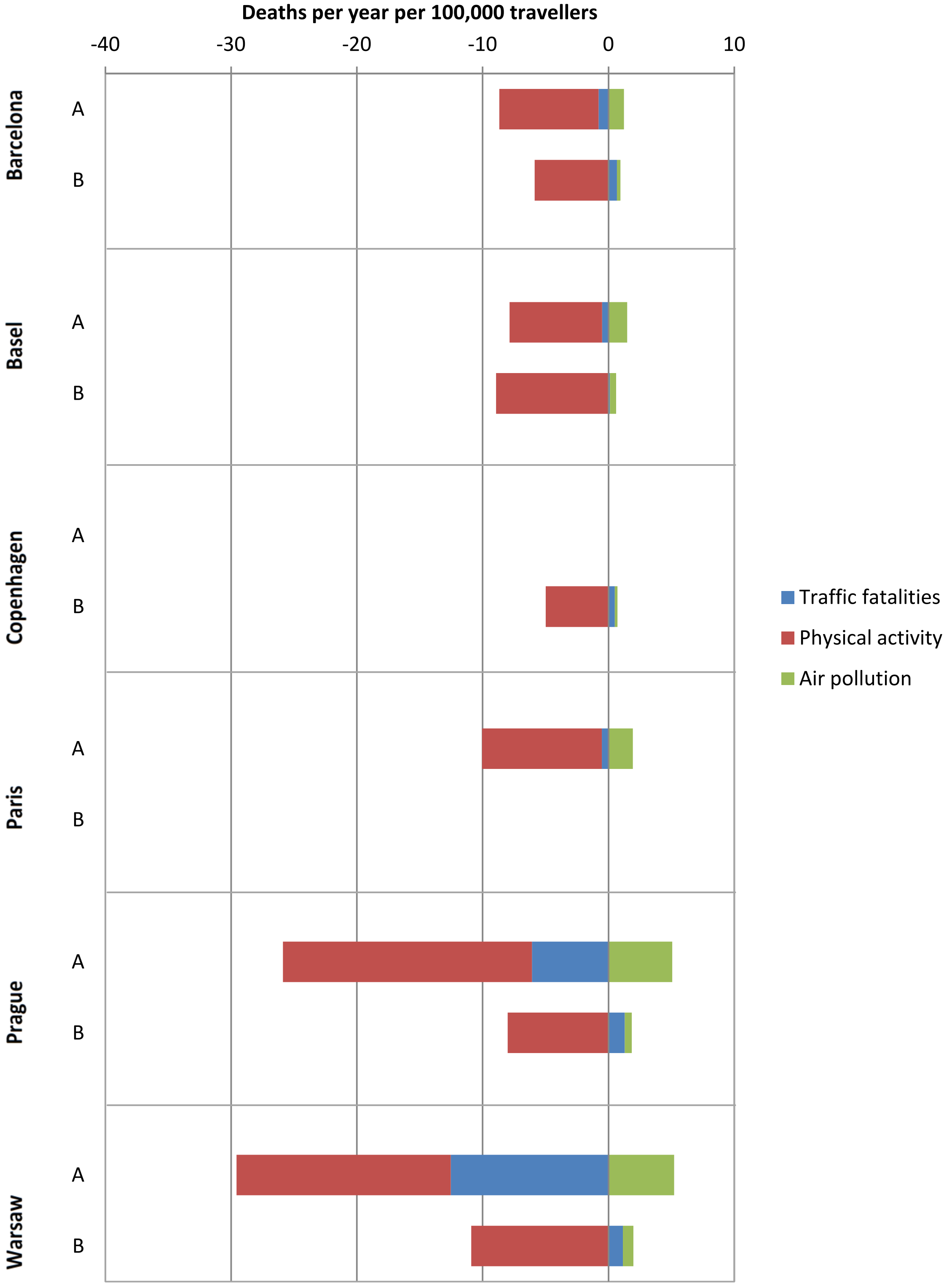

Scenario A: 35% of all trips by bicycle; Scenario B: 50% of all trips walking. The "safety in numbers" approach, was based on the incidence rate ratio of fatal traffic accidents (in cyclists or pedestrians) estimated by the six cities data.

Table 17. Number of deaths avoided or postponed **per year per 100,000 travellers (95% confidence intervals) by scenario and city (related to physical activity, air pollution and road traffic fatalities) applying to all the cities Copenhagen's death rate per kilometre travelled by bike for scenario A and Paris' pedestrian deaths rate per kilometre travelled for scenario B.**

**Scenario**	**Barcelona**	**Basel**	**Copenhagen**	**Paris**	**Prague**	**Warsaw**
**Main result (CI)**						
**A**	-7·1<br>(-4, -10)	-5·5<br>(-3, -9)	-	-6·5<br>(-3, -11)	-13·8<br>(-6, -23)	-19·6<br>(-13, -28)
**B**	-4·7<br>(-3, -7)	-7·7<br>(-5, -11)	-3·1<br>(-1, -5)	-	-3·4<br>(-1, -6)	-3·8<br>(-1, -8)
**Sensitivity analysis , applying deaths rate per km travelled of reference city (CI)**						
**A**	-7·4<br>(-5, -11)	-6·6<br>(-4, -10)	-	-8·4<br>(-5, -13)	-16·0<br>(-8, -25)	-12·9<br>(-6, -21)
**B**	-4·2<br>(-2, -7)	-6·9<br>(-4, -10)	-3·8<br>(-2, -5)	-	-6·3<br>(-4, -9)	-9·1<br>(-6, -13)

Scenario A: 35% of all trips by bicycle; Scenario B: 50% of all trips walking. Negative numbers (-) mean avoided deaths; Positive numbers mean increased deaths. CI: Confidence intervals.

Fig 7. Number of deaths per year by 100,000 travellers by scenario, health exposure and city, applying to all the cities Copenhagen's death rate per kilometre travelled by bike for scenario A and Paris' pedestrian deaths rate per kilometre travelled for scenario B.

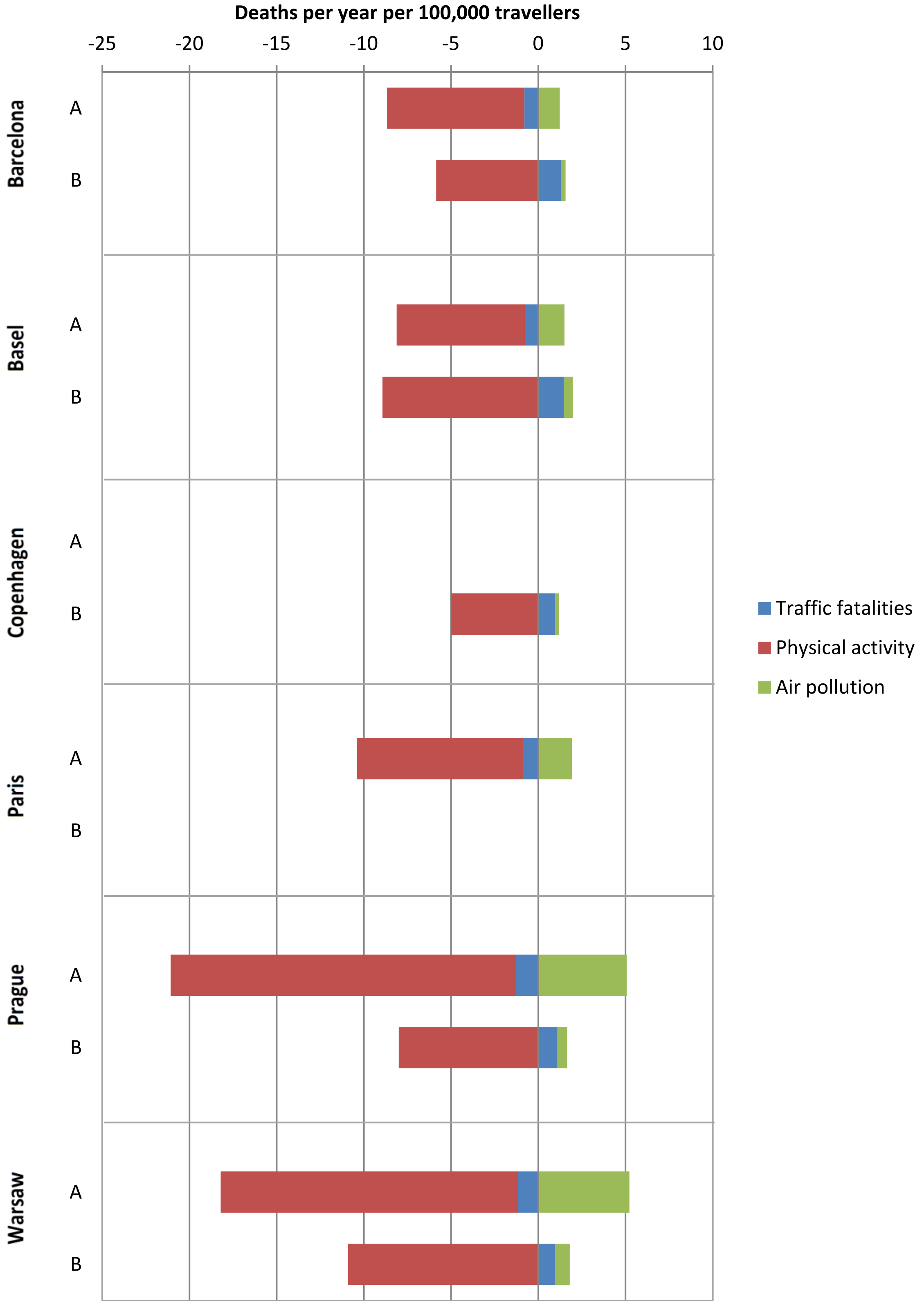

Scenario A: 35% of all trips by bicycle; Scenario B: 50% of all trips walking.

3.4. Air pollution.

Table 18. Number of deaths avoided or postponed **per year per 100,000 travellers (95% confidence intervals) by scenario and city (related to physical activity, air pollution and road traffic fatalities), assuming a dose response function of air pollution (PM2.5) and all-cause mortality (1.07 per 5 μg/m^3) derived from the ESCAPE project (European Study of Cohorts for Air Pollution Effects).**

Scenario	Barcelona	Basel	Copenhagen	Paris	Prague	Warsaw
**Main result (CI)**						
**A**	-7·1<br>(-4, -10)	-5·5<br>(-3, -9)	-	-6·5<br>(-3, -11)	-13·8<br>(-6, -23)	-19·6<br>(-13, -28)
**B**	-4·7<br>(-3, -7)	-7·7<br>(-5, -11)	-3·1<br>(-1, -5)	-	-3·4<br>(-1, -6)	-3·8<br>(-1, -8)
**Sensitivity analysis, applying ESCAPE dose response function (CI)**						
**A**	-5·6<br>(-1, -10)	-3·8<br>(1, -9)	-	-4·0<br>(1, -11)	-7·5<br>(6, -23)	-13·1<br>(0 , -28)
**B**	-4·4<br>(-2, -7)	-7·1<br>(-3, -11)	-3·0<br>(-1, -5)	-	-2·7<br>(0, -6)	-2·7<br>(1, -8)

Scenario A: 35% of all trips by bicycle; Scenario B: 50% of all trips walking. ESCAPE project reference: Beleen R, et al, 2014(6). Negative numbers (-) mean avoided deaths; Positive numbers mean increased deaths. CI: Confidence intervals.

Fig 8. Number of deaths per year by 100,000 travellers by scenario, health exposure and city, assuming a dose response function of air pollution (PM2.5) and all-cause mortality (1. 07 per 5 μg/m³) derived from the ESCAPE project (European Study of Cohorts for Air Pollution Effects).

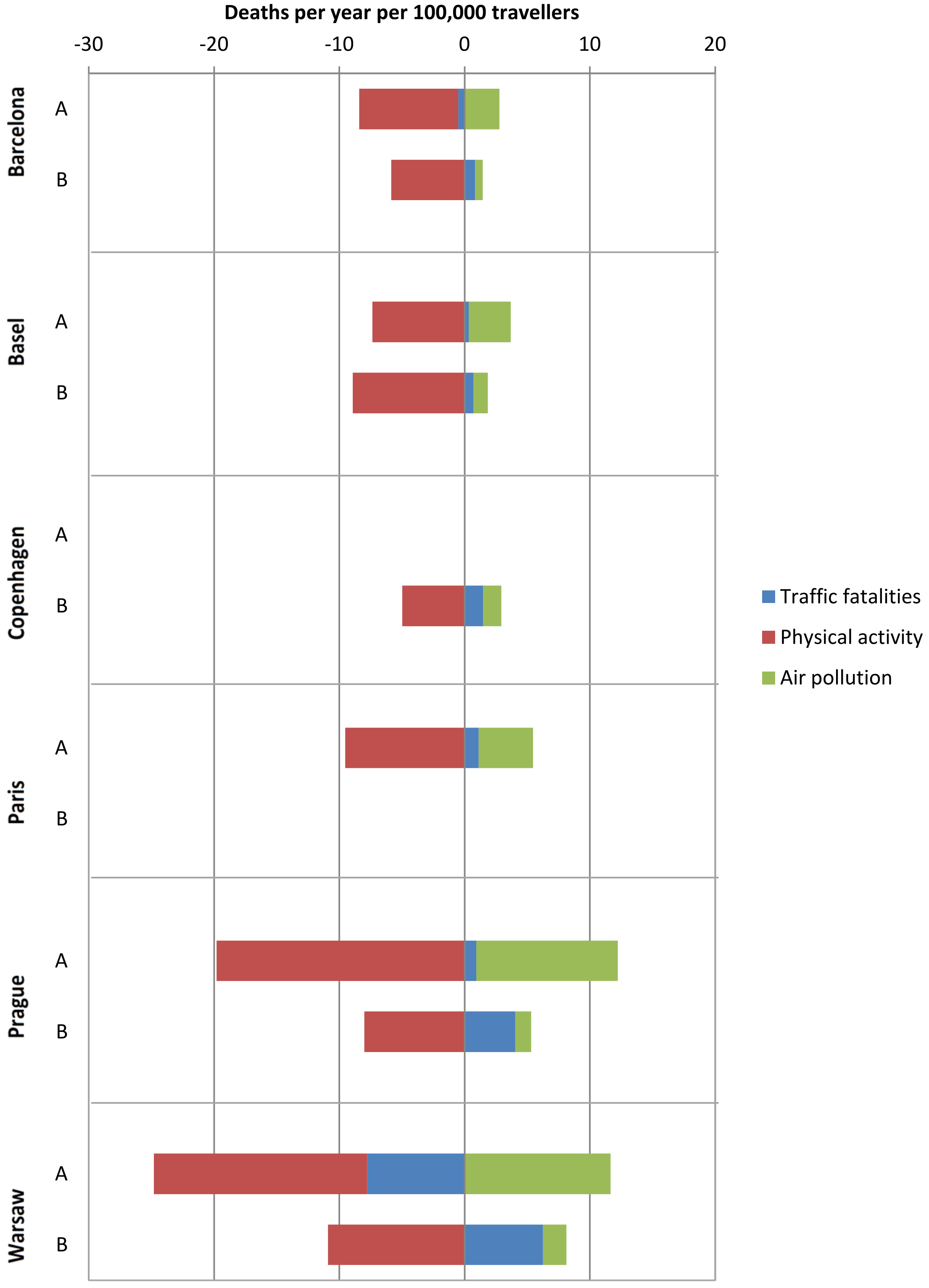

Scenario A: 35% of all trips by bicycle; Scenario B: 50% of all trips walking. ESCAPE project reference: Beleen R, et al, 2014(6).

This sensitivity analysis considered that traffic is the most important source of air pollution in the cities and assumed that traffic sources have a fivefold higher toxicity for air pollution, as suggested by previous authors(7;8). For cities like Prague, Warsaw and Paris, which have the highest concentrations of air pollution of the six cities, this fivefold toxicity factor produced small net harms rather than benefits in scenarios A (for Paris and Prague) and B (in Prague and Warsaw). For scenario A (in Paris and Prague) this is due to the high concentrations of air pollution in both cities and the substitution by cycling trips that implies higher inhalation rates in travellers compared with other modes of transport. For scenario B (in Prague and Warsaw) this is due to the combination of the high traffic fatality rates and high concentrations of air pollution in both cities.

Table 19. Number of deaths avoided or postponed **per year per 100,000 travellers (95% confidence intervals) by scenario and city (related to physical activity, air pollution and road traffic fatalities), assuming a 5 fold times more toxicity of air pollution.**

Scenario	Barcelona	Basel	Copenhagen	Paris	Prague	Warsaw
**Main result (CI)**						
**A**	-7·1<br>(-4, -10)	-5·5<br>(-3, -9)	-	-6·5<br>(-3, -11)	-13·8<br>(-6, -23)	-19·6<br>(-13, -28)
**B**	-4·7<br>(-3, -7)	-7·7<br>(-5, -11)	-3·1<br>(-1, -5)	-	-3·4<br>(-1, -6)	-3·8<br>(-1, -8)
**Sensitivity analysis, applying 5 fold times more toxicity of PM2·5 (CI)**						
**A**	-2·2<br>(1, -7)	0·3<br>(4, -5)	-	1·1<br>(6, -5)	5·5<br>(18, -10)	0·4<br>(12, -14)
**B**	-3·6<br>(-1, -6)	-5·7<br>(-2, -10)	-2·4<br>(-1, -4)	-	-1·1<br>(1, -5)	-0·5<br>(3, -5)

Scenario A: 35% of all trips by bicycle; Scenario B: 50% of all trips walking. Negative numbers (-) mean avoided deaths; Positive numbers mean increased deaths. CI: Confidence intervals.

Fig 9. Number of deaths per year by 100,000 travellers by scenario, health exposure and city, assuming a 5 fold times more toxicity of air pollution.

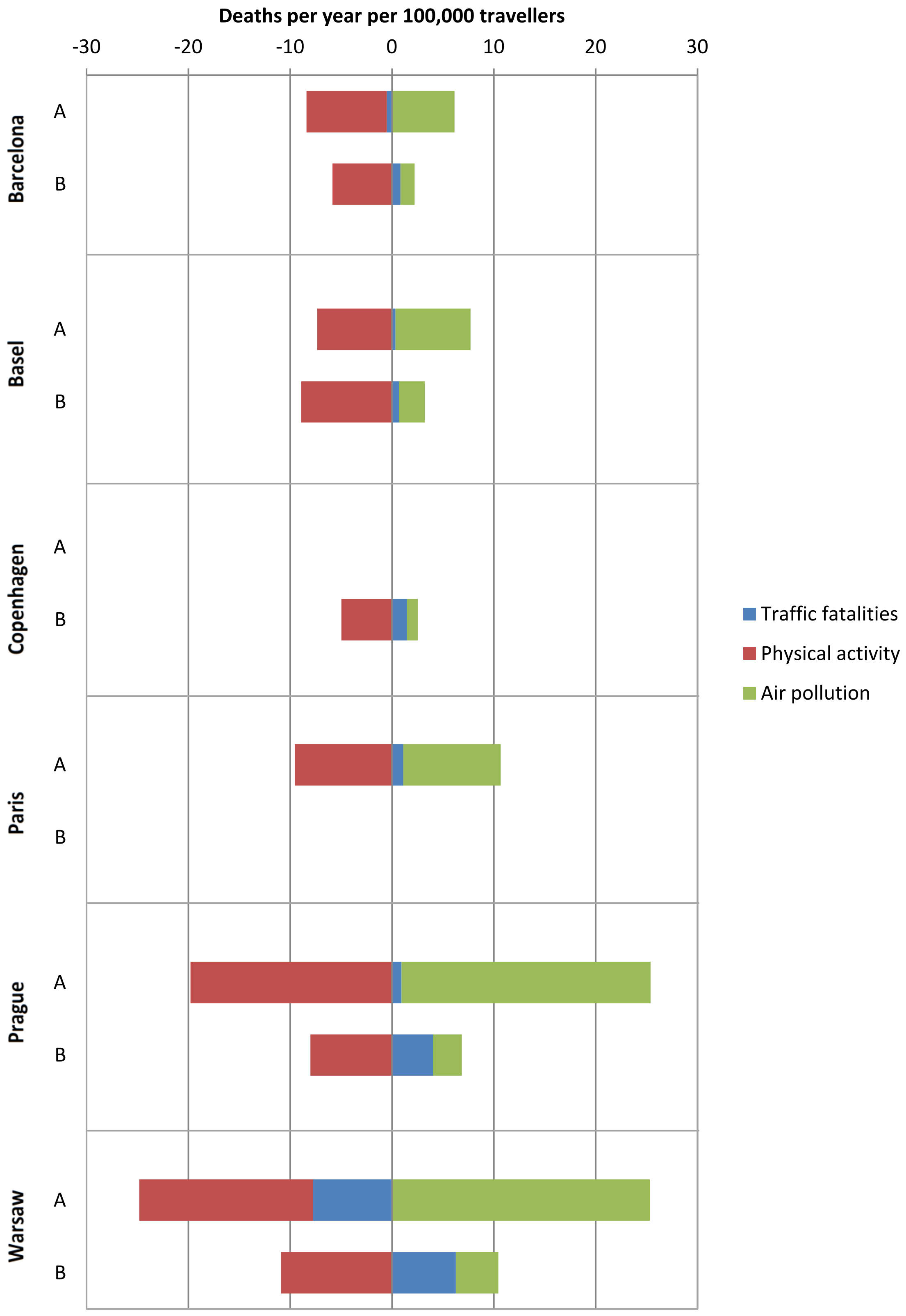

Scenario A: 35% of all trips by bicycle; Scenario B: 50% of all trips walking.

References

1. ERSO. Traffic Safety Basic Facts 2010. European Road Safety Observatory. 2010. Directorate-General for Mobility and Transport, European Commission.

2. de Nazelle A, Fruin S, Westerdahl D, Martinez D, Ripoll A, Kubesch N et al. A travel mode comparison of commuters' exposures to air pollutants in Barcelona. Atmospheric Environment 2012;59:151-9.

3. Ainsworth BE, Haskell WL, Whitt MC, Irwin ML, Swartz AM, Strath SJ et al. Compendium of physical activities: an update of activity codes and MET intensities. Med.Sci.Sports Exerc. 2000;32(9 Suppl):S498-S504.

4. Woodcock J, Franco OH, Orsini N, Roberts I. Non-vigorous physical activity and all-cause mortality: systematic review and meta-analysis of cohort studies. Int.J.Epidemiol. 2010.

5. Kahlmeier S, Cavill N, Dinsdale H, Rutter H, Götschi T, Foster C, Kelly P, Clarke D, Oja P, Fordham R, Stone D, and Racioppi F. Health economic assessment tools (HEAT) for walking and for cycling. Methodology and user guide. Economic assessment of transport infrastructure and policies. 2011. Copenhagen, World Health Organization Europe.

6. Beelen R, Stafoggia M, Raaschou-Nielsen O, Andersen ZJ, Xun WW, Katsouyanni K et al. Long-term exposure to air pollution and cardiovascular mortality: an analysis of 22 European cohorts. Epidemiology 2014;25(3):368-78.

7. De Hartog J, Boogaard H, Nijland H, Hoek G. Do the health benefits of cycling outweigh the risks? Environ.Health Perspect. 2010;118(8):1109-16.

8. Laden F, Neas LM, Dockery DW, Schwartz J. Association of fine particulate matter from different sources with daily mortality in six U.S. cities. Environ.Health Perspect. 2000;108(10):941-7.
